# Nanotechnology-assisted treatment of pharmaceuticals contaminated water

**DOI:** 10.1080/21655979.2023.2260919

**Published:** 2023-09-26

**Authors:** Amandeep Saroa, Amrit Singh, Neha Jindal, Raj Kumar, Kulvinder Singh, Praveen Guleria, Raj Boopathy, Vineet Kumar

**Affiliations:** a Department of Chemistry, Sri Guru Teg Bahadur Khalsa College, Sri Anandpur Sahib, India; b Department of Physics, Sri Guru Teg Bahadur Khalsa College, Sri Anandpur Sahib, India; c Department of Chemistry, DAV College, Bathinda, India; d Department of Chemistry, School of Basic and Applied Sciences, Maharaja Agrasen University, Baddi, India; e Department of Chemistry, DAV College, Chandigarh, India; f Department of Biotechnology, DAV University, Jalandhar, India; g Department of Biological Sciences, Nicholls State University, Thibodaux, LA, USA; h Department of Biotechnology, School of Bioengineering and Biosciences, Lovely Professional University, Phagwara, Punjab, India

**Keywords:** Pharmaceuticals, wastewater treatment, functionalized nanocomposites, adsorption, photocatalysis, metal organic frameworks (MOF)

## Abstract

The presence of pharmaceutical compounds in wastewater due to an increase in industrialization and urbanization is a serious health concern. The demand for diverse types of pharmaceutical compounds is expected to grow as there is continuous improvement in the global human health standards. Discharge of domestic pharmaceutical personal care products and hospital waste has aggravated the burden on wastewater management. Further, the pharmaceutical water is toxic not only to the aquatic organism but also to terrestrial animals coming in contact directly or indirectly. The pharmaceutical wastes can be removed by adsorption and/or degradation approach. Nanoparticles (NPs), such as 2D layers materials, metal-organic frameworks (MOFs), and carbonaceous nanomaterials are proven to be more efficient for adsorption and/or degradation of pharmaceutical waste. In addition, inclusion of NPs to form various composites leads to improvement in the waste treatment efficacy to a greater extent. Overall, carbonaceous nanocomposites have advantage in the form of being produced from renewable resources and the nanocomposite material is biodegradable either completely or to a great extent. A comprehensive literature survey on the recent advancement of pharmaceutical wastewater is the focus of the present article.

## GRAPHICAL ABSTRACT

**Figure:**
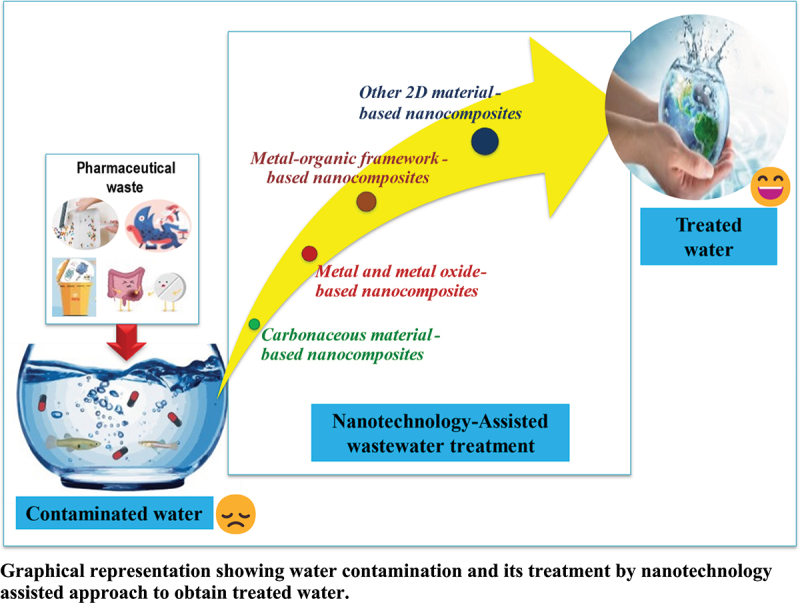

## 1. Introduction

The demand for food, and medicines is continuously increasing due to rapid increase in the global population [1]. Every country is promoting industrialization and focusing on increased production to deal with this problem. It is polluting the air, water, soil, and food items thus affecting human health. Despite the benefits of use of textile, paper pulp, paint, and plastic materials for essential human needs, it causes water contamination through discharge of volatile organic compounds and or other pollutants during dyeing, finishing of products, and paper board formation [2]. Pharma industry is helpful to cure diseases that save lives, but ultimately it also leads to environmental contamination [3]. With an increase in the rate of drugs production, pharmaceutical waste contaminants are also increasing day-by-day. Pharmaceutical pollution is majorly caused by the release of pharmaceutical drugs and their metabolites in water bodies. Pharmaceutical contaminant is a class of biomedical waste that include discarded medicines, unused creams, drug containing animal waste from hospitals, and other drugs [4]. Furthermore, in the last few years, antibiotics, anti-depressants, and anti-hypertensive drugs have been extensively used around the world due to the high prevalence rate of antiviral diseases [5]. Our body uses only 20–30% of the drug; the rest is excreted through urine and contaminates the environment [6]. Recently, it has been reported that the Cauvery River and its tributaries in India were contaminated with anti-hypertensives, anti-inflammatory, antibiotics, stimulants, and antidepressant drugs [7,8]. In this context, the drug contaminants recovered from water bodies were diclofenac, ibuprofen, isoprenaline, caffeine, carbamazepine, cetirizine, and ciprofloxacin. In addition, heavy metal ions, pesticides, plastics materials, and personal care products, were also found to be major water pollutants and their concentration in drinking water resources is increasing continuously [9]. Different conventional methods such as coagulation, reverse osmosis, filtration, evaporation, and sedimentation are used to treat water contaminated with drugs. However, these methods are unable to eradicate them completely and have low sustainability, long processing time, and high operational cost. Various researchers try to explore nanotechnology to ensure the complete removal of pharmaceutical contaminants from wastewater [10]. Nano-sorbents, nano-photocatalysts, and nano-sensors have been extensively used for the detection and removal of pharmaceutical contaminations [11]. Nanocomposites such as two-dimensional (2D) layer nanomaterials; metal chalcogenides, graphene nanosheets, boron nitride, graphitic carbon nitride g-C_3_N_4_, metal oxide nanorods [12], nanoflowers [13], and nano-leaves [14] have successfully been used for the adsorption of pharmaceutical pollutants [15]. The adsorption capacity of nano-adsorbents depends upon their surface area, the charge on the surface, porous nature, and shape of the nanomaterials [16]. Nano-photocatalysts use semiconductor nanomaterial for the UV or visible light mediated degradation of pharmaceuticals in contaminated water. Recently, the Z-scheme and dual z scheme are the most advanced photocatalytic techniques that are being explored for water treatment [17]. The benefits of using Z-scheme photocatalysts such as low electron-hole recombination rate, complete degradation, cost-effective, and visible-light driven photocatalytic response makes them more efficient as compared to conventional photocatalyst. Bismuth-based BiOX (where X=Cl, Br, I), BiVO_4_, Bi_2_S_3_, Bi_2_O_3_, Bi_2_Se_3_, Bi_2_WO_6_/CeO_2_, Fe_3_O_4_/CeO_2_/BiOI, and BiOCl@CeO_2_ have been used for the degradation of various types of antibiotics. The advantage of using bismuth and graphitic carbon nitride (Energy band gap = 2.7) eV in photo-heterostructures is their visible light-tuneable energy bandgap, low toxicity, cost-effectiveness, and high storage stability. Various types of graphene-based composites have been used for the treatment of antibiotic contaminant water. TiO_2_–ZnO/CS–Gr nanocomposite has been used to achieve the light-mediated degradation of tetracycline [6,18,19].

Nanocomposites have earlier been reported for the removal of more than one type of pharmaceutical molecules from wastewater. In such a study, maltodextrin/reduced graphene and maltodextrin/reduced graphene/copper oxide have been used for the adsorption-based removal of amoxicillin antibiotic as well as the anti-inflammatory and painkiller drug diclofenac [20]. The degradation of pharmaceutical contaminants has also been achieved at large volume in a reactor using α-Fe_2_O_3_-TiO_2_ nanocomposite on exposure to sunlight [21]. ZnFe_2_O_4_@UiO-66 nanocomposite has been used for the photo-catalytic degradation of metronidazole antibiotic [22]. The nanocomposites with magnetic properties make the separation and reusability of photo catalysts and adsorbents easy. Various types of biopolymer-based composites have been used for the treatment of pharmaceutical waste as discussed in detail elsewhere [23]. In this review, we have discussed treatments of pharmaceutical pollutants using degradation or adsorption techniques. We summarized the photocatalytic and adsorption characteristics, synthesis strategies and variation parameters of carbonaceous nanomaterials, metal organic frameworks (MOF’s) and 2D layer nanomaterials. Recently discovered carbonaceous nanomaterials such as carbon nanotubes, activated carbon, carbon quantum dots, graphene oxide and graphitic carbon nitride have been increasingly used for wastewater treatment due to their high thermal stability, low moisture sensitivity and highly tuneable surface properties [12,13]. Similarly, 2D layer nanomaterials (metal chalcogenides, graphene nanosheets, boron nitride, graphitic carbon nitride g-C_3_N_4_, metal oxide nanorods, nanoflowers, and nano-leaves) have been reviewed which are widely used in the manufacturing of visible light-assisted photocatalyst and nano adsorbents due to its high carrier mobilities, good thermal conductivity, mechanical flexibility, and high visible light absorption capacity. The novelty of this work is focused on the comparative discussion about the photocatalytic and adsorption capacity of the three most explored carbonaceous nanomaterials, metal organic frameworks (MOF’s) and 2D layer nanomaterials. It also highlights all the difficulties in handling, creating, and implementing nanocarriers for contaminated water treatment. This review article provides crucial information to address the pressing issue of pharmaceutical contamination.

## 2. Carbonaceous material-based nanocomposite for treatment of pharmaceutical wastewater

### 2.1. Carbonaceous material-based photocatalysts

Recently, attention has been focused on employing nanotechnology-based employing carbon nanocomposites because of low fabrication cost, high surface area, and environmental stability [24–26]. Due to their alluring benefits, including their customizable electronic structure, high stability, and ease of synthesis, functionalized carbonaceous nanocomposites have many applications including the treatment of pollutants. Among these carbonaceous nanocomposites, 2D graphite carbon nitride (g-C_3_N_4_) is studied widely due to suitable band energy, better stability, and high surface area [27–29]. Poor absorption of light, faster recombination of electron-hole pairs generated on exposure to light, and poor electric conductivity restricts the activity of g-C_3_N_4_ [30]. It has been documented that the functionalization of graphite carbon nitride with other substances can enhance the capabilities of g-C_3_N_4_ for the treatment of pharmaceutical waste through better photocatalytic activity [31]. Zhang et al. reported the synthesis of AgI/g-C_3_N_4_ composite for diclofenac treatment [32]. The degradation of the composite was 43.2 and 12.5 times higher than pure g-C_3_N_4_ and AgI NPs, respectively.

Jiang et al. have developed a Z-scheme photocatalyst (NiCo_2_O_4_/g-C_3_N_4_-Nvac) that uses nickel cobaltate NPs embedded in nitrogen-defects containing graphitic carbon nitride nanosheets. The defects were responsible for the activation of key species peroxymonosulfate, ^•^SO_4_^−^ based oxidation and Z-scheme-charge-transfer thus leading to better tetracycline degradation in case of the composite than NiCo_2_O_4_/g-C_3_N_4_ and g-C_3_N_4_ [33]. Likewise, Mo^6+^/Mo^5+^ oxidation-reductioncycle and PMS activation mediated degradation of tetracycline hydrochloride have been reported using MoO_3_/Bi_2_O_3_/g-C_3_N_4_ composites [34].

Guo et al. [35] reported the degradation of sulfamethoxazole using g-C_3_N_4_@PDA/BiOBr photocatalyst. The composite caused complete sulfamethoxazole degradation while other photocatalysts have shown lower photocatalytic abilities in order: g-C_3_N_4_/BiOBr (71%) > g-C3N4@ PDA + BiOBr (47%) > BiOBr (42%) > g-C_3_N_4_ (30%). The increased efficiency of the composite was attributed to the better electron transfer ability of polydopamine between g-C_3_N_4_ and BiOBr. It was established that h^+^ and ^•^O^2−^ were the main reactive species responsible for oxidizing sulfmethoxazole.

**Figure 1.**
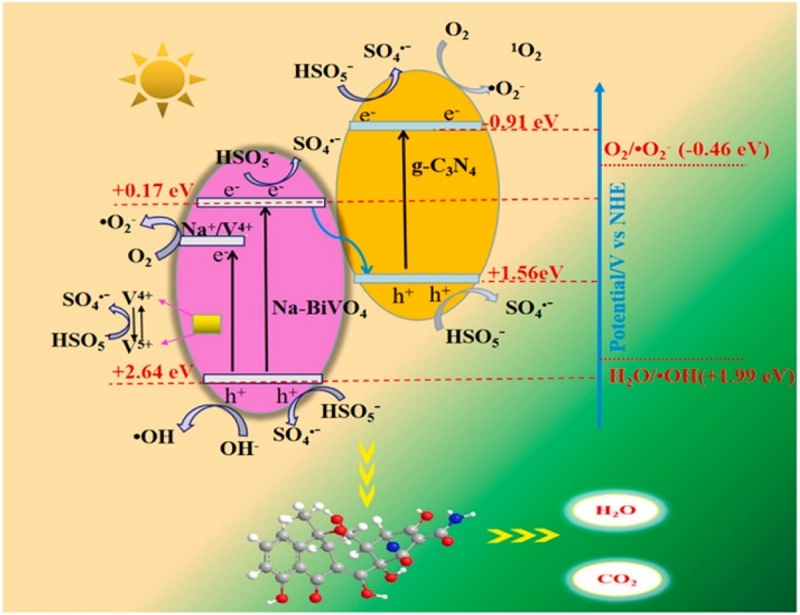
Possible mechanism of peroxymonosulfate assisted degradation of tetracycline using g-C_3_N_4_/Na-BiVO_4_ nanocomposites on exposure to light [44].

Quaternary nanohybrid photocatalyst of composition g-C_3_N_4_/NiO/ZnO/Fe_3_O_4_ has been used for the degradation of esomeprazole [36]. Esomeprazole degrades up to 95.05 ± 1.72% which is related to the synergistic effects of the various moieties. Improved charge separation, increased production of hydroxyl free radicals from H_2_O_2_, as well as a Fenton-related process, were mainly responsible for the degradation of esomeprazole. Zhang et al. prepared a Fe_3_O_4_/CdS/g-C_3_N_4_ photocatalyst using the mono-dispersion approach with 81% degradation efficiency for ciprofloxacin [37]. The separation of photogenerated carriers in photocatalytic reactions is promoted by efficient inhibition of carrier recombination. The addition of g-C_3_N_4_ broadens the optical response spectrum of CdS. With the aid of simulated solar light, the ternary catalyst CuO/CuFe_2_O_4_/g-C_3_N_4_, decomposed 99% of tetracycline hydrochloride and removed 74% of the chemical oxygen requirement. Both the values are reportedly greater than those of either CuO/CuFe_2_O_4_ and pure g-C_3_N_4_ [38]. With the aid of simulated solar light, this composite has decomposed tetracycline hydrochloride to a 99% extent and removed 74% of the chemical oxygen requirement, both of which are reportedly greater than those of either CuO/CuFe_2_O_4_ and pure g-C_3_N_4_. The enhanced reaction sites are provided by g-C_3_N_4_ substrate and inhibition of recombination of photo-excited electrons and holes is due to the synergetic effect of individual moieties in composite. ^•^O_2_^−^, h^+^, and ^•^OH were the oxidative components involved in the breakdown. Hydrothermal approach is successfully used to fabricate Fe_3_O_4_/CeO_2_/g-C_3_N_4_ composites for the photocatalytic degradation of tetracycline hydrochloride [39]. The composite having 1:0.75:0.75 mass ratio displayed an excellent degradation rate of 96.63% within 180 min. The Fenton-like reaction mechanism was followed for the degradation of tetracycline hydrochloride. g-C_3_N_4_ decreased the release of Fe from the catalyst surface and ensures proper interaction of tetracycline hydrochloride with ^•^OH. The experiment to capture active species demonstrated that ^•^OH was the main reactive species to carry out the photocatalytic degradation of the antibiotic molecule. Fe_3_O_4_/g-C_3_N_4_/MoO_3_ nanocomposite displayed 94% degradation efficiency for tetracycline degradation under visible light irradiation [40]. Nanocomposite has shown 5–20 times higher photocatalytic activity than that of the individual components owing to ^•^O_2_^−^ generation and synergetic effect of components to form Z-scheme structure leading to separation of e/h^+^ pairs.

g-C_3_N_4_/NaBiO_3_ nanocomposites produced through the hydrothermal method have been reported for the degradation of tetracycline [41]. The Z-scheme heterojunction-mediated electric field to induces charge separation and give carriers with high redox potential the opportunity to produce ^•^O_2_^−^ and ^•^OH, which reduced tetracycline to 87% of its original strength. The ternary photocatalyst BiOI/g-C_3_N_4_/CeO_2_ prepared by calcination and hydrothermal treatment has been used for the degradation of tetracycline under visible-light irradiation [42]. Tetracycline is decomposed with an efficiency of 91.6% when using a catalyst with 3 wt% BiOI/g-C_3_N_4_/CeO_2_. The photocatalytic activity was reported in order BiOI/g-C_3_N_4_/CeO_2_> BiOI/g-C_3_N_4_> BiOI > CeO_2_> g-C_3_N_4_. The nanocomposite possessed approximately 13 and 34 times higher degradation rate and rate constant as compared to g-C_3_N_4_, respectively. The better separation of produced electron-hole and dual charge transfer process between different components at ternary heterojunction were the main reasons behind the improved photocatalytic activity. Akbarzadeh et al. reported g-C_3_N_4_/Ag/AgCl/BiVO_4_-based composite for the photocatalytic degradation of ibuprofen. The hydrothermal method was used for the fabrication of g-C_3_N_4_/Ag/AgCl/BiVO_4_ composites and maximum 94.7% ibuprofen degradation was observed within 60 min of light exposure [43]. Photogenerated holes (h^+^) were the major reactive species causing the photocatalytic effect. The composite has shown better photocatalytic activity than BiVO_4_, Ag/AgCl/BiVO_4_, and g-C_3_N_4_/BiVO_4_ due to higher absorption of visible light and availability of electron-hole pairs generated along the heterojunction for a longer time. A similar mechanism was followed in the case of g-C_3_N_4_/Ag/AgCl/BiVO_4_-induced photocatalytic degradation of tetracycline. Nanocomposite has shown the best photocatalytic activity as compared to individual components and it was found to decrease in order g-C_3_N_4_/Ag/AgCl/BiVO_4_, Ag/AgCl/BiVO_4_, g-C_3_N_4_/BiVO_4_, BiVO_4_, g-C_3_N_4_. Kang et al. reported g-C_3_N_4_/Na-BiVO_4_ heterojunction as a catalyst for tetracycline degradation on visible light exposure in the presence of peroxymonosulfate [44]. About 98.20% of the tetracycline is degraded in 40 min, which is 3.13 times higher than the degradation rate by BiVO_4_. The increased tetracycline degradation over the composite is attributed to the creation of Z-scheme heterojunctions, and oxygen vacancies brought on by Na^+^ which make it easier for electron–hole couples to move apart and form free radicals as shown in Figure 1.

AgSCN/Ag_3_PO_4_/C_3_N_4_ heterojunction has been used for the decomposition of ibuprofen under sunlight. The protection of Ag_3_PO_4_ and the effective separation of electron–hole pairs by AgSCN and C_3_N_4_ components in the composite has resulted in its better degradation efficiency. The composite followed Z-mechanism and has 1.5–3.3 times higher catalytic activity than AgSCN/Ag_3_PO_4_, and Ag_3_PO_4_ [45]. Cellulose acetate (CA) membrane functionalized with Cu_2_(OH)_2_CO_3_/g-C_3_N_4_ heterojunction (Cu/CN) has been used for the degradation of tetracycline [46]. The Cu/CN@CA composite membrane has 48.43 mg/g tetracycline adsorption efficiency. Adsorption was supported by electron generation and exchange between g-C_3_N_4_ and Cu_2_(OH)_2_CO_3_ components. During solar light stimulation, type II heterojunction transfer pathway increased the membrane oxidation thus producing many active species that leads to tetracycline degradation.

**Figure 2.**
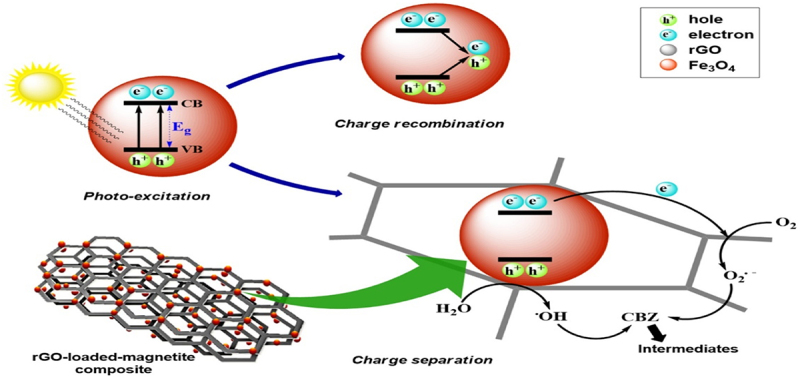
Schematic presentation of mechanism of oxidative species generation in case of rGO-loaded-magnetite composite of during the light-induced degradation process [50].

Graphene oxide (GO) is one more carbonaceous substance that is frequently used as a design template and support for many hybrid composites. The addition of GO to composite materials makes an excellent photo catalyst because it activates the fabricated composite under sunlight and maintains effective charge carrier separation because of its large surface area [47–49]. These characteristics have led to its use or coupling with other materials to maximize its benefits. Moztahida et al. [50] reported heterogeneous Fenton-like hybrid composites Fe_3_O_4_/rGO for the degradation of carbamazepine (Figure 2). The integration of rGO into the composite has the following goals: to boost the adsorption capacity of hybrid composite, decrease the aggregation of Fe_3_O_4_ nanoparticles, and decrease the charge recombination of exposed reactive sites. Within 180 min, carbamazepine is degraded by 98.7% with 10 wt% rGO loading. The composites could maintain the catalytic activity for up to five cycles in an acidic environment as it is magnetically separable and simple to regenerate.

Composite comprising 2D rGO nanosheet/3D Fe_2_O_3_/2D g-C_3_N_4_ nanosheet has been fabricated using hydrothermal process for the tetracycline and ciprofloxacin treatment [51]. The distinct heterojunction helped to produce numerous channels that caused fast exchange of light produced charge carriers which in turn significantly reduced the recombination rate. The nanocomposite has shown about 22- and 16-times higher tetracycline and ciprofloxacin degradation as compared to pristine g-C_3_N_4_ nanosheets, respectively. Su et al. fabricated Fe_3_O_4_@SiO_2_@BiFeO_3_/rGO composite using a hydrothermal technique which shows 95.6% and 98.2% ciprofloxacin and tetracycline hydrochloride degradation, respectively [52]. rGO has a crucial role in reducing the energy gap, and suppression of the recombination of charge carriers thus regulating overall photocatalytic activity. GO@Fe_3_O_4_/ZnO/SnO_2_ nanocomposite has been reported for azithromycin degradation with a maximum of 90% efficiency on exposure to UV-C [53]. Azithromycin degradation was performed in a constant bed column, and it was dependent on variables including height of the bedding material, rate of azithromycin flow, and initial concentration of azithromycin.

Carbon quantum dots (CQDs) have been regarded as remarkable candidate for interfacial modulation because of their inherent up-conversion photoluminescence, superior electron-transfer capabilities, and favorable biocompatibility [54,55]. As a result, these zero-dimensional carbon-based nanomaterials are potential candidate for photocatalysis and are a promising solid-state electron mediator. N-CQDs/oxygen vacancies (OV)-BiOBr nanocomposite prepared through hydrothermal approach has been employed for tetracycline degradation [56]. The photo response region is greatly expanded, and the charge flow is effectively directed by the synergetic action of doped QDs and OV. Additionally, oxygen vacancies and N-CQDs promoted two-electron oxygen reduction and the production of ^•^O_2_^−^ and H_2_O_2_ through a single electron, respectively, which resulted in direct oxidation and indirect photo-Fenton-like reaction of tetracycline. With the increase in the amount of N-CQDs in composite, the photocatalytic performance is greatly improved.

Likewise, CQDs included in the CQDs/BiOCOOH/ultrathin g-C_3_N_4_ nanosheets (uCN) composite has mediated the transfer of electrons between BiOCOOH and uCN to fasten photocatalytic degradation of sulfathiazole. The composite containing 50 wt% uCN leads to complete degradation after 90 min of LED lamp exposure due to quick transfer and separation of photo-generated charges. The degradation efficiency was reported to be 97.35% ± 1.57 after five cycles of reuse [57]. CQDs synthesized from corncob biomass using photoinduced approach have been incorporated to form Fe_3_O_4_/BiOBr/CQDs composite. The composite has been used for the photo-catalytic degradation of carbamazepine. The photocatalytic effect was mainly caused by Z-scheme structures formation, photoluminescence of CQDs, the synergetic effect of CQDs and Fe_3_O_4_ and generation of ^•^O_2_^−^, h^+^, and ^•^OH [58]. Table 1 enlists details about different carbonaceous material-based nanocomposites that have been explored as photo-catalyst for the remediation of pharmaceuticals wastewater.

**Table 1.** Carbonaceous material-based nanocompositess as photo-catalyst for remediation of pharmaceuticals wastewater.

S. No.	Carbonaceous material-based nanocomposite	Synthesis method	Pharmaceutical pollutant	Pollutant dose (mg/L)	Catalyst dose;(mg/L)	Source of light; capacity	Degradation efficiency (%) percentage	Irradiation time (min)	Reference
1	AgI/g-C3N4	Deposition-precipitation	Diclofenac	1	10	Xenon lamp; 300 W	100	80	[32]
2	NiCo2O4/g-C3N4-Nvac	Ultrasonic	Tetracycline hydrochloride	25	10	Solar light	97.4	30	[33]
3	MoO3/Bi2O3/g-C3N4	—	Tetracycline hydrochloride	40	30	Solar light	98	140	[34]
4	g-C3N4@PDA/BiOBr	Solvothermal	Sulfamethoxazole	2.5	30	Xe lamp; 300 W	100	60	[35]
5	g-C3N4/NiO/ZnO/Fe3O4	Hydrothermal	Esomeprazole	30	700	White LED bulb; 23 W	95	70	[36]
6	Fe3O4/CdS/g-C3N4	Mono-dispersion	Ciprofloxacin	20	50	Xenon lamp; 250 W	81	330	[37]
7	CuO/CuFe2O4/g-C3N4	Calcination	Tetracycline hydrochloride	20	100	Xenon lamp; 300 W	99		[38]
8	Fe3O4/CeO2/g-C3N4	Hydrothermal	Tetracycline hydrochloride	50	50	Xenon lamp; 300 W	96.63	180	[39]
9	Fe3O4/g-C3N4/MoO3	Calcination	Tetracycline	40	10	Xenon lamp; 1000 W	94	102	[40]
10	g-C3N4/NaBiO3	Hydrothermal	Tetracycline	25	50	Xe lamp; 300 W	87.1	30	[41]
11	BiOI/g-C3N4/CeO2	Hydrothermal	Tetracycline	20	50	Xenon lamp; 300 W	91.6	120	[42]
12	g-C3N4/Ag/AgCl/BiVO4	Hydrothermal	Ibuprofen	2	250	Fluorescent lamp; 8W	94.7	60	[43]
13	g-C3N4/Na-BiVO4	Hydrothermal	Tetracycline	20	200	Xenon lamp; 300 W	98.20	40	[44]
14	AgSCN/Ag3PO4/C3N4	Calcination	Ibuprofen	5	50	Halide lamp; 500 W	91	6	[45]
15	Cu2(OH)2CO3/g-C3N4/cellulose acetate (Cu/CN@CA)	Nonsolvent-induced phase separation	Tetracycline	–	–	Solar light	–	120	[46]
16	Fe3O4/rGO	Co-precipitation	Carbamazepine	5	500	Solar light	98.7	180	[50]
17	rGO/Fe2O3/g-C3N4	Hydrothermal	Tetracycline; ciprofloxacin ciprofloxacin	50	100	Halogen lamp; 500 W	98;97	60	[51]
18	Fe3O4@SiO2@BiFeO3/rGO	Hydrothermal	Tetracycline hydrochloride; ciprofloxacin ciprofloxacin	10	1000	Xenon lamp; 500 W	98.2;95.6	90	[52]
19	GO@Fe3O4/ZnO/SnO2	In-situ mixing	Azithromycin	30	100	UV-C lamp; 6 W	9.06	120	[53]
20	N-CQDs/OV-BiOBr	Hydrothermal	Tetracycline	20	50	Xe lamp; 300 W	89.42	60	[56]
21	CQDs/BiOCOOH/uCN	Ultrasonically dispersion	Sulfathiazole	10	800	LED lamp; 50 W	99.28	90	[57]
22	Fe3O4/BiOBr/CQDs	Photoinduced	Carbamazepine	10	30	LED bulb; 50 W	99.52	120	[58]

### 2.2. Carbonaceous material-based adsorbents

**Figure 3.**
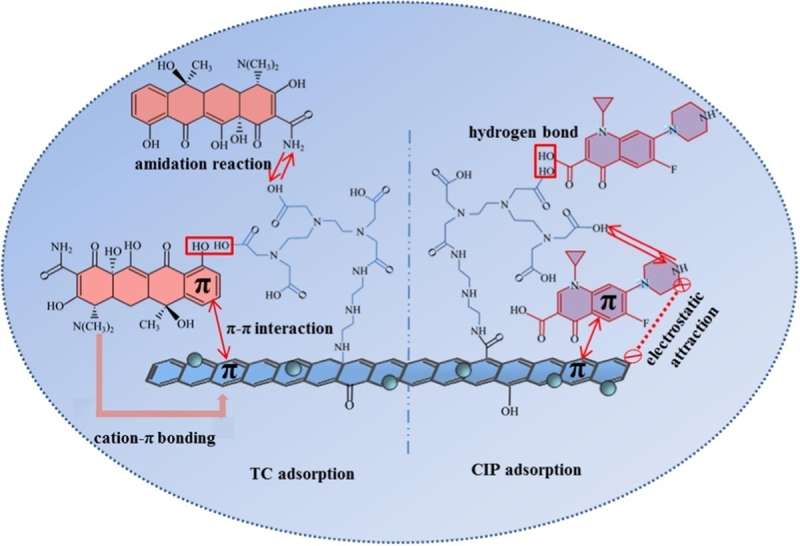
Adsorption schematic diagram of tetracycline and ciprofloxacin on DDMGO [61].

Adsorption offers greater benefits in terms of pollutant treatment strategies when compared to photo degradation and other membrane-based technologies, because it is an efficient, straightforward, ecologically beneficial, and simple procedure [59]. The efficacy of adsorption for antibiotic removal depends up on the sorbent nature, such as specific surface area, porosity, and pore diameter. Graphene has a polarized surface that can interact with various type of hydrophobic organic contaminants with the help of π-π interaction. However, it is difficult to separate and extract graphene from a suspension after adsorption in large-scale environmental applications. The problem can be overcome by further functionalization of magnetic graphene oxide with compounds rich in amino and carboxyl groups, among other things [60]. Li et al. documented the use of graphene oxide functionalized with diethylenetriamine-pentaacetic acid (DDMGO) for the adsorption of tetracycline and ciprofloxacin [61]. The abundance of oxygen or nitrogenous groups on the surface, high surface area to volume ratio, and the ease with which it could be easily separated and recycled due to magnetic properties from aqueous solution are some of the factors that make nanocomposites a better option as compared to individual components. In binary systems, the coexisting competing antibiotics had a higher inhibitory effect on tetracycline sorption than ciprofloxacin. Tetracycline and ciprofloxacin were removed by DDMGO mostly through the amidation process, H-bonds, and π-π interaction (Figure 3). Cation-π and electrostatic interaction could be used to explain tetracycline and ciprofloxacin uptake, respectively. DDMGO was documented to possess better capability for removal of tetracycline and ciprofloxacin adsorption capabilities than other reported adsorbents namely, graphene nanosheets, functionalized graphene oxide with magnetic properties, MWCNTs, and activated carbon, removal [62–65].

rGO-Fe_3_O_4_ has been fabricated for phenazopyridine residue removal from waste-water samples [66]. The magnetic nanocomposite has 14.064 mg/L sorption capacity at pH = 6 for phenazopyridine with maximum of 91.4% phenazopyridine adsorption efficiency. Phenazopyridine adsorption occurs due to accelerated charge transport at the interface of nanocomposite components and its interaction with aromatic rings and oxygen-terminated group on the nanocomposite surface.

NPs can be functionalized using enzyme to increase their adsorption efficiency. Chitosan-graphene oxide (CsGOn) composite functionalized with horseradish peroxidase (HRP) has been used for the removal of ofloxacin [67,68]. HRP forms a covalent bond with HRP-CsGOn thus increasing the mechanical strength of the enzyme leading to ofloxacin removal [69]. This green nanocomposite has shown 378 mg/g maximum adsorption efficiency and can be recycled for at least five cycles. Fe_3_O_4_@SiO_2_-Chitosan/GO nanocomposite is explored for tetracycline elimination [70]. The greatest tetracycline adsorption capacities on nanocomposite with and without Cu(II) were 183.47 and 67.57 mmol/kg, respectively. The electrostatic and π–π interactions between the tetracycline and Cu (II) components of the composite were the major factors involved in the adsorption process.

Fe_3_O_4_@ polyethyleneimine (PEI)-rGO nanocomposite synthesized using a self-assemble method has been documented for adsorption of polar non-steroidal anti-inflammatory medicines; ketoprofen, naproxen, diclofenac, and ibuprofen. This composite involved the synergetic effect of both PEI and rGO which demonstrated greater extraction efficiency as compared to individual components, Fe_3_O_4_@ PEI, Fe_3_O_4_-rGO, and Fe_3_O_4_@PEI-GO. The adsorption mechanism displayed the potential of Fe_3_O_4_@PEI-rGO as a polar and π-π type adsorbent [71]. Similarly, magnetically Fe_3_O_4_@ graphene nanoplatelets composite prepared by solid-phase fabrication has been reported for the removal of these non-steroidal anti-inflammatory drugs. H-bonding, hydrophobic, π–π electrostatic, and *n*–π electron acceptor–donor interaction were responsible for adsorption of drugs on the surface of nanocomposite [72].

Tetracycline elimination rate of 99.4% is observed by using MnO_2_/graphene nanocomposite [73]. The incorporation of MnO_2_ nanorods has modified adsorption efficiency as it avoided stacking too many graphene sheets together throughout the adsorption process and gave additional chances to tetracycline molecules for contacting the adsorbents. The primary adsorption mechanism involved complex formation by Mn(IV) and π-π interactions involving benzene ring of graphene sheets with tetracycline. Nanocomposite containing 40% MnO_2_/graphene has shown 198 mg g^−1^ adsorption capacity that was better than free treated graphene sheets with 170 mg g^−1^ capacity. A triple-network composite hydrogels with composition carbon nanotubes/L-cysteine@graphene oxide/sodium alginate (CNTs/L-cys@GO/SA) has been reported to remove ciprofloxacin antibiotic from aqueous contaminated samples [74]. The internal three-dimensional space of network structure has shown maximum adsorption of 181 mg/g for ciprofloxacin in a weak acidic environment.

The ability of graphitic carbon nitride material to selectively interact with the various type of pollutant has been extended to remove pharmaceutical molecules from contaminated waste. It offers other advantages like being a nonmetallic environmentally safe material with tuneable thermal, chemical, and physical properties [75,76]. Fe_3_O_4_-g-CN@PEI-β-CD nanocomposite removes tetracycline with an efficiency of 833.33 mg/g. Larger surface area, more porosity, and presence of function groups that form nanocomposite–drug complex were main factors responsible for tetracycline adsorption [77].

The activated carbon (AC) at the microscale is frequently utilized as a sorbent to remove various contaminants because of its unique structure and strong adsorption ability [78]. AC made from the biomass is extremely effective as an adsorbent for removing pharmaceutical molecules. This includes hormone, antibiotics, anti-inflammatory, and analgesics compound [79–81]. Nanoscale AC due to its high surface area, affordable commercial availability, and variety of surface-active groups support the binding and hence, the removal of pollutant [82]. Activated carbon-Fe_3_O_4_ (AC-Fe_3_O_4_) nanocomposite with magnetic properties has been fabricated for the removal of promazine which is a phenothiazine antipsychotic drug. Nanocomposites possess high adsorption efficiency, quick adsorption, and simple magnetic separation. Complete drug removal with 101.01 mg/g adsorption capacity was achieved within 6 min [83]. Yeast-extracted carbon functionalized with magnetic NPs (NP-YC) prepared from ethanol industry waste has been used for the Ibuprofen removal. Ibuprofen was removed from sewage effluent with 51 mg/g capacity by NP-YC [84]. Nanocomposite made of carbon obtained from plantain peel functionalized with zinc oxide NPs (PPAC-ZnO) has been used for the adsorption of chloroquine, a medication used to treat malaria and COVID-19 [85]. Maximum of 78.89% chloroquine adsorption was achieved.

Spherical fullerene is one of the members of the carbon nanomaterials family that has zero-dimensional structure with exceptional electron withdrawing capabilities [86]. These nanostructures have properties that are on the cusp of being in between nanomaterials and molecules [87]. Yet, numerous initiatives from the field of materials science to a variety of applications on fullerene chemistry and nano-molecular chemistry resulted in the development of readily produced composites [88]. Fullerene produces active oxygen that helps in wastewater treatment [89]. Functionalized magnetic fullerene nanocomposite (FMFN) has been used for the adsorption of the antibiotic Ciprofloxacin. Nanocomposite is produced by the catalytic thermal degradation of recyclable poly(ethylene terephthalate) bottle wastes as resource using ferrocene as a catalyst with magnetite precursor [90]. After five cycles, the adsorbent’s capacity for regeneration is reported to remain high. The reported description about carbonaceous material-based nanocomposites as adsorbent for remediation of pharmaceuticals wastewater is tabulated in Table 2.

**Table 2.** Carbonaceous material-based nanocomposites as adsorbent for remediation of pharmaceuticals wastewater.

S. No.	Carbonaceous material-based nanocomposite	Synthesis method	Pharmaceutical pollutant	Adsorbate dose (mg/L)	Adsorbent dose	Adsorption capacity (mg/g)	Time (min)	Surface area (m^2^/g)	Reference
1	DDMGO	—	Tetracycline; Ciprofloxacin	50	2.28	294.12 111.73	1440	176	[61]
2	rGO-Fe_3_O_4_	Co-precipitation	Phenazopyridine	800	800	14.06	5	213	[66]
3	Fe_3_O_4_@SiO2-Chitosan/GO	—	Tetracycline	400	50 ml	183.47 mmol/kg	240	—	[70]
4	Fe_3_O_4_@PEI-RGO	Self-assemble	Ketoprofen; naproxen; diclofenac; ibuprofen	50	5	—	5	—	[71]
5	MnO_2_/graphene	Hydrothermal	Tetracycline	200	50	198	1500	106	[73]
6	CNTs/L-cys@GO/SA	Emulsion template	Ciprofloxacin	200		181	3600		[74]
7	Fe_3_O_4_@GNP	Solid-phase fabrication technique	Ibuprofen; ketoprofen; naproxen; diclofenac sodium salt	20	—	7.63 8.76 10.6 14.3	20	355.5	[72]
8	Fe_3_O_4_-g-CN@PEI-β-CD)	—	Tetracycline	265	—	833.33	29	57.12	[77]
9	AC/Fe_3_O_4_	—	Promazine	40	10	101.01	6	788.66	[83]
10	NP-YC	Coprecipitation	Ibuprofen	2.5	120	120	—	823	[84]
11	PPAC-ZnO	Precipitation	Chloroquine	—	100	881.586	40	606.07	[85]
12	FMFN	—	Ciprofloxacin	65	500	—	153	336.84	[90]

## 3. Metal and metal oxide-based nanocomposites for treatment of pharmaceutical wastewater

### 3.1. Metal and metal oxide-based photocatalyst

Functionalization of metal/metal oxide nanocomposite for wastewater remediation is well documented. Functionalized metal/metal oxide nanocomposite has been reported to contain high photocatalytic activity, low band gap, better separation of charge carriers, and recovery of the catalyst. The details about metal and metal oxide-based nanocomposite for remediation of pharmaceutical wastewater are tabulated in Table 3. CuO/ZnO composite prepared using thermal process has been used for visible light driven ciprofloxacin degradation [91]. Hydroxyl radical (•OH) and the oxidative hole (h^+^) generation by nanocomposite were mainly responsible for the ciprofloxacin photodegradation.

**Table 3.** Metal and metal oxide-based nanocomposite as photocatalyst for remediation of pharmaceutical wastewater.

S. No.	Metal and metal oxide-based nanocomposite	Synthesis method	Pharmaceutical pollutant	Pollutant dose	Catalyst dose	Light source	Degradation percentage	Irradiation time	Reference
1	CuO/ZnO	—	Ciprofloxacin	10 ppm	1 g/L	500 W Xe lamp	—	30 min	[91]
2	ZnO/Ag_2_CO_3_/Ag_2_O	—	Ibuprofen	50 ppm	0.1 g	100 W LED light	99.3	—	[92]
3	Ag/AgBr/ZnFe_2_O_4_	In-situ growth	Carbamazepine	10 ppm	1 g/L	93.38 W LED light	52.8	240 min	[93]
4	BiOBr/Fe_3_O_4_@SiO_2_	Solvothermal	Ibuprofen	2 mg/L	1 g/L	Fluorescence lamp	100	60 min	[94]
5	Ce@TiO_2_	Hydrothermal	Diclofenac	—	50 mg	—	—	80 min	[95]
6	Cu-TiO_2_@SWCNT	Hydrothermal	Sulfamethazine	30 mg/L	0.9 g/L	Solar light	—	135 min	[96]
7	Fe_3_O_4_/Bi_2_S_3_/BiOBr	Solvothermal	Diclofenac Ibuprofen	10 mg/L	0.03 g	50 W LED lamp	93.81 96.78	40 min	[97]
8	PPy-ZnO	Polymerization	Diclofenac	10 mg/L	1 g/L	Xenon lamp	81%	60 min	[98]
9	TiO_2_@ZnFe_2_O_4_/Pd	Photodeposition	Diclofenac	10 mg/L	0.03 g/L	Solar light	86.1%	86.1	[99]
10	ZnFe_2_O_4_/SiO_2_/TiO_2_	In-situ mixing	Etodolac	15 mg/L	0.05 g	300 W xenon lamp	100	20 min	[100]
11	SDS/BiOBr-MB	Hydrothermal	Tetracycline Ciprofloxacin	20 mg/L 10 mg/L	25 mg	300 W xenon lamp	85 95	120 min	[101]
12	FeNi_3_@SiO_2_@ZnO	In-situ mixing	Tamoxifen	10 mg/L	0.01 g/L	500 W LED lamp	100	60 min	[102]
13	Fe_3_O_4_/ZnO	Polyol mediated	Sulfamethoxazole Trimethoprim Erythromycin Roxithromycin	100 mg/L	100 µg/L	UV-A lamp	100 94 95 98	240 min	[103]
14	ZnFe_2_O_4_@TiO_2_/Cu	Solvothermal	Naproxen	15 mg/L	0.04 g/L	Sunlight	80.73	120 min	[104]
15	TiO_2_/Fe_2_O_3_	Ultrasonic-assisted sol gel synthesis	Paracetamol	50 mg/L	0.1 g/L	450 W mercury vapor	87.8	90 min	[105]
16	Fe_3_O_4_/BiVO_4_/CdS	In-situ mixing	Tetracycline	10 mg/L	100 mg	300 W xenon lamp	87.37	90 min	[106]
17	Fe_3_O_4_@SiO_2_@Bi_2_O_2_CO_3_-sepiolite	Hydrothermal	Ciprofloxacin, Tetracycline hydrochloride	10 mg/L	50 mg	300 W xenon lamp	92.1 100	90 min	[107]

A simple two-step synthesis approach has been used to fabricate ZnO/Ag_2_CO_3_/Ag_2_O for the photocatalytic degradation of ibuprofen. The presence of Ag_2_CO_3_ and Ag_2_O heterostructure over the ZnO surface led to 99% degradation of Ibuprofen that is 10-fold better degradation than the pure ZnO [92]. By employing Ce@TiO_2_ nanocomposites under UV irradiation, diclofenac photocatalytic degradation of the drug is increased as reported [95]. After being exposed to UV light for 80 min, the diclofenac underwent full degradation. Comparing the effectiveness of Ce@TiO_2_ nanocomposites to commercial TiO_2_, nano TiO_2_, and CeO_2_ revealed that these materials have increased activity. Until to the fifth cycle, the Ce@TiO2 nanocomposite remained stable. Payan et al. produced Cu-TiO_2_@functionalized single-walled carbon nanotubes for the catalytic degradation of sulfamethazine within 135 min [96]. On the surface of the SWCNTs, Cu-doped TiO_2_ particles are reported to be distributed uniformly that help to uniformly capture light induced electrons and reduction of electron–hole recombination.

Polypyrrole (PPy)-ZnO composite fabricated using polymerization technique has been used for the diclofenac degradation. PPy-ZnO has demonstrated a higher photocatalytic rate constant which is about twice as compared to ZnO. The h^+^ was the primary oxidizing species in charge of the 81% degradation of diclofenac by PPy-ZnO in just 60 min [98]. Fe_3_O_4_/Bi_2_S_3_/BiOBr nanocomposites prepared using Fe_3_O_4_ nanosphere, Bi_2_S_3_ nanorod, and BiOBr nanosheet demonstrated excellent photo catalytic performance for the removal of diclofenac and ibuprofen with removal efficiencies of 93.81% and 96.78%, respectively [97]. The solvothermal method used to prepare nanocomposite result has been found to improve the crystal behavior that was mainly responsible for better photocatalytic degradation by nanocomposite as compared to the pure individual component. h^+^ and e^−^ played the most important roles in the diclofenac and ibuprofen photo degradation as per active species trapping experiment. BiOBr/Fe_3_O_4_@SiO_2_ nanocomposite prepared via the solvothermal method has been reported for the photocatalytic degradation of ibuprofen [94]. The nanocomposite was a mesoporous magnetic photocatalyst that can achieve almost complete mineralization within 360 min of visible light exposure. The two main pathways for degradation are e^−^_CB_-mediated processes and direct-hole oxidation. The magnetic property was useful in photocatalyst recycling and approximately 80% of the photocatalytic activity was maintained even after five consecutive cycles.

Model pharmaceutical pollutant has been used to study photocatalytic removal using TiO_2_@ ZnFe_2_O_4_/Pd nanocomposite. The nanocomposite has been used in batch and continuous systems to carry out diclofenac degradation using sunlight [99]. Pd coated nanocomposite displayed better diclofenac photo degradation than ZnFe_2_O_4_ and TiO_2_@ZnFe_2_O_4_ owing to its magnetically properties nanocomposite was recycled up to five cycles of usage, although the maximum photo catalytic activity was dropped from 86.1% in the first cycle to 71.38% in the final cycle. p-type nanoanatase TiO_2_/Zinc ferrite NPs based photocatalyst has been used for the degradation of anti-inflammatory drug, etodolac [100].

**Figure 4.**
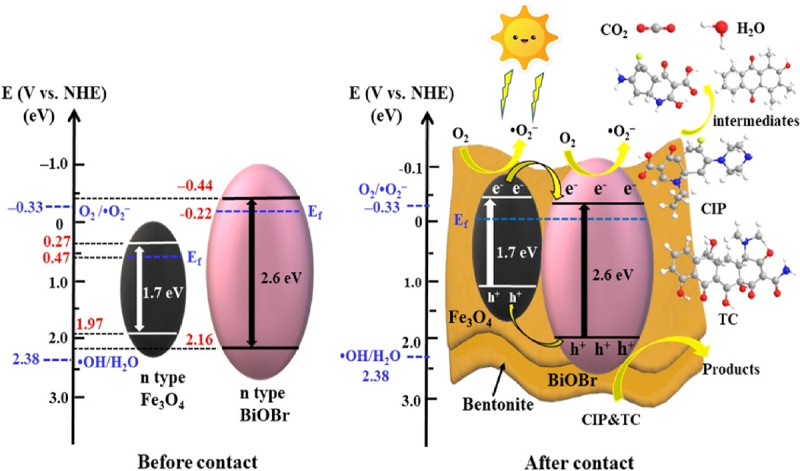
Schematic representation of the mechanism of enhanced photogradation of tetracycline and ciprofloxacin by SDS/BiOBr-MB [101].

Superparamagnetic Ag/AgBr/ZnFe_2_O_4_ nanocomposite has been reported for photocatalyic degradation of carbamazepine [93]. The photocatalyst can be easily separated using external magnetic field. The semiconductor conduction and valance bands levels were studied to reveal Z- scheme mechanism of heterostructure photocatalyst. The photocatalytic activity has been observed to increase upon the addition of persulfate and it decreases upon the addition of H_2_O_2_. Overall, ^•^O_2_^−^ and ^•^SO_4_^−^ were the main species involved in the photocatalytic process. Sodium dodecyl sulfate (SDS)/BiOBr-magnetic bentonite (MB) nanocomposites has been used for the catalytic degradation of tetracycline and ciprofloxacin [101]. Nanocomposite has shown better tetracycline and ciprofloxacin degradation activity with maximum 85% and 95% efficiency, respectively. Further, an external magnet allowed for the rapid separation of the photocatalysts from the solution within 20 s. SDS/BiOBr-MB composite showed improved photocatalytic degradation as compared to BiOBr, and BiOBr-MB. The synergetic effect due to presence of SDS, the n–n heterojunction between BiOBr and Fe_3_O_4_, and the addition of bentonite, which created a platform for dispersing the growth of BiOBr. Overall, these factors were responsible for the improved photocatalytic efficacy of SDS/BiOBr-MB (Figure 4). The photo-generated electron–hole pairs of SDS/BiOBr-MB are more effectively separated and transferred to the n-n heterojunction between BiOBr and Fe_3_O_4_. The capacity of the bentonite’s permanently negative charge to absorb photo-generated holes led to a decrease in the number of photo-generated carriers.

Nasseh et al. prepared magnetic FeNi_3_@SiO_2_@ZnO nanocomposite to photo-catalytically remove tamoxifen from wastewater simulated light [102]. The photocatalyst could also effectively complete five rounds of photodegradation in a row. In addition to this, sulfamethoxazole, trimethoprim, erythromycin, and roxithromycin are four persistent antibiotics that are being removed from surface waters using a Fe_3_O_4_/ZnO based nanocomposite [103]. The accessible Fe in the nanocomposite has a synergistic impact that aided in the secondary reaction of antibiotic oxidation by photo-Fenton. The system increased Fe_3_O_4_/ZnO photonic efficiency, simplicity of magnetic separation and catalyst reusability that depends upon the reactor configuration. Antibiotic removal efficiency was higher using stirred system as the catalyst was dispersed more evenly thus increasing the surface to volume ratio. The solvothermal method is used to generate the ZnFe_2_O_4_@TiO_2_/Cu nanocomposite which is used as photocatalytic degradation of naproxen [104]. In sunlight, the nanocomposite showed 80% naproxen photodegradation. This composite exhibited ferromagnetic property, stability and cyclability up to five cycles with maximum 72.31% removal efficiency. The nanocomposite showed superior photocatalytic activity compared to individual components. This is as a result of the high surface area of copper that increases the charge and energy transfer between metal semiconductors. Flower shaped TiO_2_/Fe_2_O_3_ core–shell artificial light driven catalysts have been reported for the degradation of paracetamol [105]. An increase in the content of TiO_2_ in composite improved the rate of paracetamol photodegradation and mineralization. The photocatalyst has strong stability and was magnetically separable up to four cycles.

To create the Fe_3_O_4_/BiVO_4_/CdS heterojunction photocatalyst, pure Fe_3_O_4_ and BiVO_4_ surfaces are simultaneously decorated with CdS in a low-temperature water bath system [106]. During visible light irradiation, FBC-3 (2 mmol of Cd(CH_3_COO)_2_·2 H_2_O) showed the highest rate of tetracycline degradation (87%). The main active groups involved in tetracycline degradation are h^+^ and •O_2_^−^. The Z-scheme transfer route as a means of enhancing photocatalytic activity is considered. Fe_3_O_4_@ SiO_2_@ Bi_2_O_2_CO_3_-sepiolite nanocomposite has been reported for ciprofloxacin and tetracycline hydrochloride photodegradation with 92% and 100%, respectively [107]. Sepiolite can increase the surface area of composite and provided a strong interfacial effect between the sepiolite and rest of the components of composite. This circumstance led to high capacity of light harvesting, separation of charge carriers, and formation of many active reaction sites on photocatalyst.

## 4. Metal-organic framework-based nanocomposites for treatment of pharmaceutical wastewater

### 4.1. Metal-organic framework-based adsorbents

MOFs have a spatial topology with a periodic porous structure and are often created by the self-assembly of organic ligand, metal ions, or clusters of metal ion. Ultra-high porosity, large surface area, tuneable structure, and ease of preparation are some of the amazing characteristics of MOFs [108]. These fascinating advantages make MOFs appealing for drug delivery [109], photocatalysis, and gas storage/separation application [110,111]. MOFs and their composites have shown tremendous application promise for wastewater treatment due to their improved water stability, great flexibility, and tuneable physicochemical features [112–115]. Some common examples that demonstrate this idea include graphene oxide/MOFs (GO/MOFs) and polydimethylsiloxane/MOFs (PDMS/MOFs) [116,117]. The incorporation of graphene oxide in GO/MOFs composites offers a hydrophobic environment that prevent water molecules from attacking the coordination bonds. PDMS/MOFs coating with PDMS can lead to the synthesis of composite with hygroscopic or water repellent properties. The various types of MOFs functionalized nanocomposites that have been explored as an absorbent for the remediation of pharmaceutical wastewater are tabulated in Table 4.

**Table 4.** Mofs based nanocomposites as absorbent for remediation of pharmaceutical wastewater.

S. No.	MOF-based nanocomposite	Synthesis method	Pharmaceutical pollutant	Adsorbate dose	Adsorbent dose	Adsorption capacity	Time	Surface area	Reference
1	MIL-101(Cr)/SA	Hydrothermal	Ibuprofen ketoprofen	2.5 g/L	—	62.6 mg/g 130.6 mg/g	180 min	910.52 m^2^/g	[117]
2	MIL-101(Cr)/CS	Hydrothermal	Ibuprofen ketoprofen	2.5 g/L	—	103.2 mg/g 156.5 mg/g	180 min	910.52 m^2^/g	[117]
3	MWCNT/MIL-53(Fe)	Solvothermal	Tetracycline hydrochloride; oxytetracycline hydrochloride; chlortetracycline hydrochloride	20 mg/L	0.2 g/L	89.21 mg/g 60.83 mg/g 43.08 mg/g	500 min	60.17 m^2^/g	[118]
4	MWCNT/NH_2_-MIL-53(Fe)	Solvothermal	Tetracycline hydrochloride; chlortetracycline hydrochloride	20 mg/L	10 mg	368.49 mg/g 254.04 mg/g	500 min	125.50 m^2^/g	[119]
5	GO/MIL-101	Microwave Synthesis	Naproxen; Ketoprofen	5.0 mg/L	5 mg	112 mg/g 171 mg/g	240	3144 m^2^/g	[120]
6	Zr/Fe-MOFs/GO	Solvothermal	Tetracycline hydrochloride	10 mg/L	50 mg	70 mg/g	4 hr	1.5061 m^2^/g	[121]
7	GNP/UiO-66 graphene nanoplatelets	Hydrothermal	Diclofenac sodium	35 mg/L	0.01 g	452.46 mg/g	40 min	910.581 m^2^/g	[122]
8	MrGO/ZIF	Hydrothermal	Methamphetamine	—	1 mg	523.12 µg/g	60 min	203.7977 m^2^/g	[123]
9	MIL-68(Al)/GO pellets	Hummer’s method	Tetracycline	60 mg/L	0.2 g/L	228 mg/g	540 min	1266.69 m^2^/g	[124]
10	MIL-101(Cr)@GO	Hydrothermal	Sulfadiazine; sulfadoxine; sulfamethoxazole	1 mg/L	50 mg	135.14 mg/g 101.01 mg/g 119.05 mg/g	60 min	3351.64 m^2^/g	[125]
11	UiO-66-(COOH)_2_/GO	Solvothermal	Tetracycline hydrochloride	10 mg/L	20 mg	164.91 mg/g	48 h	369.6 m^2^/g	[126]
12	CuCo/MIL-101)	Hydrothermal	Tetracycline	10 mg/L	50 mg	225.179 mg/g	60 min	2422.682 m^2^/g	[127]
13	Mn_2_(BDC)_2_(DMF)_2_ derivedMnO@C	Solvothermal	Chloramphenicol; Diclofenac; Tetracycline; Ciprofloxacin	10 to 40 mg/L	0.1 g/L	79.9 mg/g 92.4 mg/g 170.3 mg/g 235.6 mg/g	360 min	263 m^2^/g	[128]
14	HKUST-1 Cu/Cu_2_O/CuO@C	Solvothermal	Ciprofloxacin tetracycline	40 mg/L	0.5 g/L	67.5 112.5 mg/g	360 min	80 m^2^/g	[129]
15	Fe_3_O_4_/HKUST-1	—	Ciprofloxacin; norfloxacin	20 mg/L	2 mg	538 513	30 min	327.9 m^2^/g	[113]
16	MIL-53(Fe)/Fe_3_O_4_	Co-precipitation	Doxycycline	1000 mg/L	0.02 g	322	30 min	75.5 m^2^/g	[130]
17	Fe_3_O_4_@MIL-68 (Al)	Ultrasonically dispersion	Minocycline	50 mg/L	0.2 g/L	248.05	160 min		[131]
18	MIL-101/Fe_3_O_4_	Hydrothermal	Ciprofloxacin	15 mg/L	1–15 mg	63.28	50 min	764.4 m^2^/g	[132]
19	Fe_3_O_4_@MOF-235(Fe)	Solvothermal	Ciprofloxacin	250 mg/L	0.5 g/L	256.40	5 hr	974 m^2^/g	[133]
20	Fe_3_O_4_@MIL-100(Fe)	Solvothermal	Ciprofloxacin	250 mg/L	0.5 g/L	322.58	5 hr	2800 m^2^/g	[133]
21	ZIF-8@SiO_2_@ Fe_3_O_4_	Solvothermal	Ceftazidime	1 mg/L	0.001 g	96.84	20 min	1273.08 m^2^/g	[134]
22	ZIF-8/NH_2_-MIL-53(Al))	Hydrothermal	Doxycycline; Tetracycline; oxytetracycline; chlortetracycline	0–1000 mg/L	0.2 g/L	561 533 526 578 mg/g	300 min	1821 m^2^/g	[135]

Adsorption is a highly competitive technology to remove pharmaceutical waste from aqueous solutions due to its cost-effective nature, ease of use, and lack of chances of secondary contamination. MIL-101(Cr) with sodium alginate (MIL-101(Cr)/SA) and MIL-101(Cr) with chitosan (MIL-101(Cr)/CS) composite prepared by combining MIL-101(Cr) with SA and CS has been used as an adsorbent for adsorption of ibuprofen and ketoprofen [117]. The composite beads exhibit higher adsorption capability than individual components. The electrostatic interactions between Cr center and functional groups of the composites with the functional groups of the pharmaceutical contaminant were responsible for the adsorption. Further, it was possible to improve the hydrophobic properties by integrating adequate water-resistant functional groups in the MOFs. Xiong et al. reported MWCNT loaded iron-MOF [MIL-53(Fe)] composite for adsorption of tetracycline hydrochloride, oxytetracycline hydrochloride, and chlortetracycline hydrochloride from aqueous solutions [118]. The nanocomposite has 1.6, 1.2, and 1.4 times more pore volume, specific surface area, and pore diameter than MIL-53, respectively due to the presence of MWCNT. This contributed to enhancement in the maximum tetracycline hydrochloride, oxytetracycline hydrochloride, and chlortetracycline hydrochloride adsorption capacity of nanocomposite that was 1.25–8.28 times higher than that of MWCNT, respectively. Likewise, amino-functionalized MIL-53(Fe) containing MWCNT/NH_2_-MIL-53(Fe) composite has also been used to adsorb chlortetracycline and tetracycline hydrochloride [119]. The improved mesoporosity of MWCNT/NH_2_-MIL-53(Fe), the π-π interaction and hydrogen bonding between amino functional groups of composites with hydroxyl functional groups on tetracycline hydrochloride/chlortetracycline hydrochloride were collectively accountable for the increased adsorption capacity.

The addition of graphene oxide (GO) to MOF adsorbents produced more functional substances with wide pore structure in order to increase the adsorption efficiency. Hence, highly porous MOF composites are shaped by fusing GO with MIL-101(Cr) [120]. The linking of GO layers to the MOF framework led to enhancement thus producing more pore openings and hydrogen bonding due to the availability of diverse functional groups on the composites. Compared to pure MIL-101 and commercial activated carbon, MIL-101GO (3%) composites showed significantly better adsorption toward anti-inflammatory drugs like naproxen and ketoprofen in a aqueous solution. In a similar way, Wei et al. reported GO/MOFs using the solvothermal technique to remove tetracycline hydrochloride [121]. Zr/Fe-MOFs/GO composite has shown good absorption efficiency for tetracycline hydrochloride as compared to GBCM350 activated carbon [136], Fe_3_O_4_@SiO_2_-chitosan/GO [70], ZIF-8 [112], NH_2_-MIL-101(Cr) [137], MIL-101(HCl) [138], and UiO-66 [139]. Further, the hydrothermal approach is used to fabricate GNP/UiO-66 nanocomposite derived from zirconium-based MOF (UiO-66) and graphene nanoplatelets [122]. GNP/UiO-66 NPs have a higher BET surface area than GNP NPs (750–910.518 m^2^/g), which raised their maximum adsorption effectiveness to 99.02%. MrGO/ZIF composite is created for the adsorption of methamphetamine by attaching ZIF-67 on magnetic substrates [123]. Some of the properties contributed by different components of nanocomposites were responsible for its drug adsorption performance that include large surface area to volume ratio, hierarchical pore structure, Van der Waals forces, electrostatic interactions, and H-bonding and unsaturated coordinate bonds formation. Additionally, aluminum-based MOF/GO (MIL-68(Al)/GO) composite has been reported for aqueous tetracycline adsorption with maximum 228 mg/g capacity [124]. In addition to intricate hydrogen bonding, π–π stacking and Al-N covalent bond formation were prominent interactions that supported the adsorption process. MIL-101(Cr)@GO has also been investigated for the adsorption of three different sulfonamides, namely sulfadiazine, sulfadoxine, and sulfamethoxazole [125]. It is found to be reusable upto five cycles with a maximum 101–135 mg/gadsorption capacity. Hydrophobic interaction, π−π interactions, hydrogen, and coordinate bond formation are driving forces for the adsorption of sulfonamides on nanocomposite. Zr(VI)-based MOF UiO-66-(COOH)_2_/GO nanocomposite have shown five times better tetracycline hydrochloride adsorption than that of UiO-66 [126]. It has achieved increased adsorption active sites by incorporating carboxyl groups and GO. Weak electrostatic interaction, chemical coordination, and π–π interaction between nanocomposite and tetracycline hydrochloride accounted for the adsorption process.

**Figure 5.**
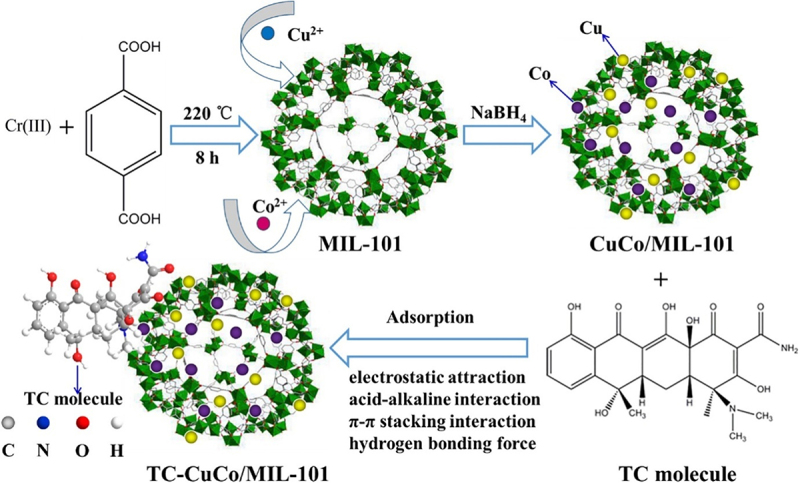
Schematic diagrams of the synthesis of CuCo/MIL-101 and the adsorption of tetracycline [127].

The metal doping of MOFs changes the electronegativity of the composite surface which ultimately alters the electrostatic interactions between the nanocomposite and pharmaceuticals. Jin et al. [127] reported the formation of CuCo/MIL-101 composite by loading bimetallic CuCo NPs onMIL-101 for the removal of tetracycline from aqueous solutions. Nanocomposites have 140% more tetracycline removal efficiency than pure MIL-101. Improvement in adsorption efficiency was attributed to electrostatic interactions between CuCo NPs and tetracycline molecules as illustrated in Figure 5.

Porous carbon materials can be functionalized to acquire essential properties including structural stability, porosity, and functional groups so that they can be used as excellent adsorbents. MnO@C nanocomposite produced by pyrolysis of metals or metal oxides self-supported on porous carbon was used for the adsorption of antibiotic pollutants including tetracycline, ciprofloxacin, diclofenac, and chloramphenicol in aqueous medium [128]. Adsorption of antibiotics occurs due to the presence of graphitic carbon, Mn-O and other bonds in the nanocomposite. Additionally, Cu/Cu_2_O/CuO@C were reported for the adsorption of ciprofloxacin and tetracycline [129]. Nanocomposites have a porous hollow structure along with several surface chemical bonds on its surface leading to antibiotic adsorption. XPS spectroscopy revealed the involvement of Cu (I, II) oxidation states, hydrogen bonds, π–π, and *n*–π interactions for higher adsorption efficiency than individual components.

The interference observed during the practical application in term of recovery and regeneration of the adsorbent after use is difficult to manage at large scale. One way to accomplish the goal of recovering and reusing the adsorbent is by combining magnetic materials with MOFs. Based on this concept, Wu et al. developed a Fe_3_O_4_/HKUST-1 copper-based MOF composite as an efficient and recyclable adsorbent for the removal of ciprofloxacin and norfloxacin types of fluoroquinolone antibiotics [113]. Due to its high adsorption rate, magnetic composite 513–538 mg/g antibiotics adsorption capacity was achieved in less than 30 min. Nanocomposite can be separated from the contaminated water using external magnets. The primary sorption process involved in the removal of ciprofloxacin and norfloxacin from water using Fe_3_O_4_/HKUST-1 included π–π interactions, hydrophobic interactions, electrostatic interactions, and hydrogen bonding. Fe_3_O_4_/HKUST-1 was effectively regenerated and found reusable up to more than 10 times. Naeimi and Faghihian prepared MIL-53(Fe)/Fe_3_O_4_ composite for the removal of 322 mg/g doxycycline from aqueous solutions [130]. The super paramagnetic character of composite was used to recycle it for six regenerations. Minocycline was removed within 160 min using magnetic aluminum-based MOF composite. Fe_3_O_4_@MIL-68 (Al) has maximum 248.05 mg/g adsorption efficiency [131]. The adsorption mechanism revealed the participation of intricate interactions, such as electrostatic adsorption, hydrogen bonding, covalent Al–N and Fe–N bonds and π–π stacking. Another magnetic composite, MIL-101/Fe_3_O_4_, has been reported for the adsorptive removal of ciprofloxacin [132]. Temperature-dependent difference in adsorption capacity was obtained with maximum 63.28 mg/g at 298 K. By exploiting the advantage of easily phase separable magnetic sorbent over non-magnetic sorbent, Fe_3_O_4_@MIL-100(Fe) and Fe_3_O_4_@MOF-235(Fe) is scrutinized for ciprofloxacin removal [133]. However, magnetic nanocomposite with Fe_3_O_4_@MIL-100 (Fe) composition has a comparatively higher 322.58 mg/g adsorption capacity. The adsorption occurred in the form of a monolayer over the nanocomposite surface. The presence of Fe_3_O_4_NPs was mainly responsible for better adsorption capacity due to an increase in the number of accessible sorption sites as compared to bare MOFs.

Zeolite imidazolate framework-8 (ZIF-8) containing MOFs are known for their exceptional chemical stability, porosity, thermal/water stability, and active sites endowed for various types of chemical and physical interactions with pharmaceutical molecules in aqueous phase [120]. Ceftazidime was removed by adsorptive means using ZIF-8@SiO_2_@Fe_3_O_4_, which was produced through the self-assembly of ZIF-8 with SiO_2_@Fe_3_O_4_ [134]. Due to hydrophobic and electrostatic interactions, as well as a high specific surface area of 1273.08 m^2^/g, it demonstrated a greater adsorption capacity of 96.84 mg/g. After five cycles, nanocomposite could be efficiently regenerated with over 90% efficiency. Additionally, a dual MOFs-based smart platform produced using ZIF-8 and 2D amino-functionalized Al-MOF (NH_2_-MIL-53(Al)) nanoplates for the adsorption and detection of doxycycline, tetracycline, oxytetracycline, and chlortetracycline [135]. In particular, the pyridine N of ZIF-8 in combination with -NH_2_ group on surface of NH_2_-MIL-53(Al) displayed significant affinity toward these drugs due to hydrogen-bond formation, π−π stacking and chemisorption.

### 4.2. Metal-organic framework-based photocatalysts

Adsorption and degradation processes for pharmaceutical waste are the main water clean-up techniques. The adsorption process could only reduce the concentration of pharmaceutical waste, but it could not get rid of this pharmaceutical waste [140]. Antibiotics are mostly degraded by electrochemical, Fenton and photo-catalytic techniques. Among these processes, photo-catalysis could save a significant amount of energy by fully utilizing solar light throughout the degradation process [141]. The development of heterojunctions is very advantageous for raising photo-catalytic performance of MOFs. It enhances the amount of light absorption by other components of heterojunction which produce more free electrons, voids, and reaction sites along with facilitating the charge separation [142]. A detailed description about MOFs derived nanocomposites as photocatalyst for remediation of pharmaceutical waste water is tabulated in Table 5.

**Table 5.** Mofs based nanocomposites as photocatalyst for remediation of pharmaceutical wastewater.

S. No.	MOF based nanocomposite	Synthesis method	Pharmaceutical pollutant	Pollutant dose	Catalyst dose	Light source	Degradation percentage	Irradiation time	Reference
1	CoTiO_3_/UiO-66-NH_2_	Hydrothermal	Norfloxacin	20 mg/L	0.02 g	300 W Xenon lamp	90.13	60 min	[143]
2	CuWO_4_/Bi_2_S_3_/ZIF67	Hydrothermal	Metronidazole; cephalexin	20 mg/L	0.3 g/L	400 W LED	95.6 90.1	80 min	[144]
3	Bi_2_O_2_CO_3_/porous g-C_3_N_4_	Calcination	Sulfamethazine	1 g/L		300 W Xenon lamp	90.31	180 min	[145]
4	AgI/UiO-66	Solvothermal	Sulfamethoxazole	5 mg/L	0.5 g/L	300 W Xenon lamp	99.6	20 min	[146]
5	Ag_2_S/MIL-53(Fe)	Solvothermal	Tetracycline	20 mg/L	0.67 g/L	300 W Xenon lamp	96	60 min	[147]
6	UiO-67/CdS/rGO	Hydrothermal	ofloxacin	10 mg/L	0.05 g/L	300 W Xenon lamp	93.4	30 min	[148]
7	MoS_2_/ZIF-8	Solvothermal	Ciprofloxacin; tetracycline	20 mg/L	0.4 g/L	300 W Xenon lamp	93.2 75.6	180 min	[149]
8	TCPP@UiO-66	Solvothermal	Diclofenac	30 mg/L	5 mg	350 W Xenon lamp	90	100 min	[150]
9	CuBi_2_O_4_@ZIF-8	Hydrothermal	Tetracycline	20 mg/L	0.5 g/L	5 W LED lamp	75.3	60 min	[151]
10	ZnO/ZIF-9	Sonocrystallization	Tetracycline	10 mg/L	0.2 g/L	UV	87.7	60 min	[152]
11	ZIF-8@TiO_2_	Hydrothermal	Tetracycline	100 mg/L	0.6 g/L	300 W Xenon lamp	92	120 min	[153]
12	MIL-101(Fe)/TiO_2_	Solvothermal	Tetracycline	20 mg/L	1 g/L	300 W xenon lamp	98.01	80 min	[154]
13	Fe_3_O_4_@MIL-100(Fe)	Microwave Synthesis	Diclofenac sodium	60 mg/L	0.1 g/L	500 W xenon lamp	99.4	180 min	[155]
14	MIL-100(Fe)@Fe_3_O_4_/CA	In-situ method	Tetracycline	10 mg/L	10 mg	150 W Xenon lamp	85	3 h	[156]
15	MIL-88A derived Fe3O4@PBS	Calcination method	Tetracycline	10 mg/L	500 mg/L	300 W Xenon lamp	97.5	45 min	[157]
16	Cu_2_O/Fe_3_O_4_/MIL-101(Fe)	In-situ method	Ciprofloxacin	20 mg/L	0.5 g/L	500 W Xenon lamp	99.2	105 min	[158]
17	MIL-101(Fe)/CoFe_2_O_4_/GO	Solvothermal	Tetracycline hydrochloride	30 mg/L	10 mg/L	100 W LED	92	50 min	[159]
18	Fe_3_O_4_@MIL-53(Fe)	Calcination Method	Ibuprofen	10 mg/L	20 mg	500 W Xenon lamp	99	60 min	[160]
19	g-C_3_N_4_@CoFe_2_O_4_/Fe_2_O_3_	Hydrothermal synthesis	Tetracycline; sulfamethoxazole; diclofenac; ibuprofen; ofloxacin	30 mg/L	10 mg	500 W Xenon lamp	99.7 94.8 97.0 96.1 96.5	80 min	[161]
20	Fe_3_O_4_@MIL-100(Fe)	In-situ method	Levofloxacin	200 mg/L	0.33 g/L	300 W Xenon lamp	93.4	180 min	[162]

CoTiO_3_/UiO-66-NH_2_ binary p–nheterojunction has been reported for the degradation of norfloxacin by designing and evolving competent heterogeneous catalyst for harvesting solar energy [143]. The inverted V-shaped Mott–Schottky plot supported this binary p–n heterojunction. The improved photocatalytic reaction followed Type-II p–n hetero junction charge transfer mechanism with 90.13% degradation. Subudhi et al. reported various advantages for this heterojunction: (i) an increase in the lifespan of photo-generated active species including a variety of active sites by heterojunction, (ii) strengthening of the hydrophilicity due to hetero atoms which provide suitable contact site between CoTiO_3_/UiO-66-NH_2_ and norfloxacin, (iii) greater ability to respond to solar light than a photocatalyst in its purest state and (iv) unlocking new surface pores to capture the suitable adsorbates. Hydrothermal approach has been used to synthesize the double Z-scheme CuWO_4_/Bi_2_S_3_/ZIF67 ternary heterostructure for the degradation of metronidazole and cephalexin under visible light illumination [144]. Comparing this ternary heterostructure with CuWO_4_/Bi_2_S_3_ and pristine ZIF67, the photoactivity of the former showed a notable improvement. Based on the dual Z-scheme structure, the augmentation of CuWO_4_/Bi_2_S_3_/ZIF67 photoactivity is attributed to the higher surface area, photo-stability, and suppression of bandgap in addition to better charge separation. Metronidazole and cephalexin, the ternary heterostructure had maximum degradation efficiencies of 95.6% and 90.1%, respectively. The ternary heterostructure showed remarkable chemical stability and reusability after six cycles. The trapping experiments are used to investigate the governing active species and the results showed that the main oxidants are hydroxyl free radicals. Bi_2_O_2_CO_3_/porous g-C_3_N_4_ Z-scheme heterojunction is created by Wang et al. to remove sulfamethazine [145]. The amount of intimate contact at the interface is greatly increased as Bi_2_O_2_CO_3_ NPs evenly disperse across surface, edge, and interlayer in g-C_3_N_4_ nanosheet. The mechanism analysis showed that Bi_2_O_2_CO_3_/g-C_3_N_4_ composites specifically encouraged molecular oxygen activation into singlet oxygen (^1^O_2_). This may be attributed to Bi-N bonds at the interface and the development of Z-scheme system which speeded up the oxidation of ^•^O_2_^−^ by holes. The composites outperformed the pristine g-C_3_N_4_ in the elimination of sulfamethazine after 90 min of visible light illumination and helped to selectively generate (^1^O_2_) in a targeted manner during molecular oxygen activation.

For the photocatalytic elimination of sulfamethoxazole, an in-situ growing approach is used to generate a visible light responsive composite photocatalyst AgI/UiO-66 composite [146]. As compared to pure AgI, the AgI/UiO-66 had significantly improved photocatalytic performance. Superoxide radicals (^•^O_2_^−^) and hydroxyl radicals (^•^OH) are shown to be the predominant active species in the photocatalytic degradation of sulfamethoxazole as per the radical capture studies. Phenyl nitrification, isoxazole ring hydroxylation, and S-N bond cleavage are all components of the sulfamethoxazole degradation pathway. Ag_2_S NPs are coupled with MOFs material (MIL-53(Fe)) using an easy solvothermal method for tetracycline degradation to create Ag_2_S/MIL-53(Fe) heterojunction hybrids [147]. Nano composites have shown higher photocatalytic activities as compared to bare individual components. The creation of heterojunction with close contact between interface improved charge transfer and separation, band gap reduction, and increased photo absorption which in turn was responsible for the increased catalytic activity. Moreover, the synergistic effects of light and Fenton catalysis allowed the addition of H_2_O_2_ which further improved the catalytic activity.

A series of mesoporous composites of UiO-67/CdS/rGO-x has been fabricated by incorporating visible-light responsive CdS NPs into MOF and encapsulating them in excellent conductor rGO thin sheets. In this composite, the simultaneous utilization of sunlight-harvesting and electron-relay-penetration is exploited [148]. It led to a maximum 93.4% ofloxacin degradation within 30 min under the simulated photo exposure. This is credited to following synergistic aspects of extension of the light absorption range of MOF, band-gap matching, and maintenance stability and activity of catalytic site. Further, TiO_2_-based catalysts upon using a molybdenum disulfide/ZIF-8 composite photocatalyst have been reported for ciprofloxacin and tetracycline hydrochloride degradation [149]. Nanocomposite has better photodegradation due to better conductivity of light generated electron and lowering the recombination rate.

MOFs can have structural defects and combining different capabilities because of heterogeneity as they are solid-state crystals. By carefully managing the structural heterogeneity and defects through defect engineering, it is possible to manipulate the desired characteristics of MOFs, such as the production of mesopores, reduction of network rigidity/density, enhancement of active sites, development of novel functionalities, and mechanical properties. In continuation to this aspect, Gao et al. reported the use of defect-engineered MOFs with multi-functionalities for the degradation of diclofenac [150]. The level of defect affected the crystallite morphology, phase purity and properties of nanocomposite with a maximum 590 mg/g adsorption capacity. The formation of ^1^O_2_was observed that ultimately indicated the involvement of type II photosensitization reaction. The degradation of diclofenac is facilitated by both the energy transfer from TCPP to triplet oxygen and the electron transport from TCPP to Zr clusters. A dual-function platform with CuBi_2_O_4_@ZIF-8 composites has been documented to carry out sensing and degradation of tetracycline [151]. Nanocomposite owing to the better formation of free radicals and charge separation was observed to cause maximum 75.2% degradation which was higher than 50–73% in case of individual components, ZIF-8 and CuBi_2_O_4_.

The extraordinary function that the heterostructure plays in charge separation made the development of heterostructures in photocatalysts an efficient way to increase their catalytic activity. Regarding this, a simple so no crystallization method is successful in producing a new type II photocatalyst ZnO/ZIF-9 heterojunction [152]. ZnO/ZIF_0.5_ could attain 87.7% tetracycline photodegradation within 60 min. Type II heterojunction formation between ZnO and ZIF-9 is primarily responsible for the higher photocatalytic activity because it increases the conductance of light generated electrons to make it easier to separate charges from holes. Li et al. reported ZIF-8@TiO_2_NPs containing composite for the catalytic degradation of tetracycline [153]. MOF with porous structure and superior adsorption capacity, electron transmission by ZIF-8 in the ZIF-8@TiO_2_ micron composite, reducing the charge carrier’s recombination rate, visible light absorption and narrow band gap are prominent factors for enhancing the catalytic ability of the composite photocatalyst.

MIL-101(Fe)/TiO_2_ composite with magnetic properties has been prepared to photodegrade tetracycline to take the advantage of easy separation of catalyst [154]. Under sun light irradiation, 92.76% of the 20 mg/L tetracycline was degraded in 10 min. Further, TiO_2_ added to the composite has a significant effect on the catalytic process, which could be enhanced by light exposure to produce significant amounts of the radicals ^•^O_2_^−^ and ^•^OH. The composite has high reusability as it is easy to recover the composite from tetracycline solution using a magnet. Using Fe_3_O_4_ as a metal precursor, Fe_3_O_4_@MIL-100(Fe) was created through microwave in 30 min and used to treat diclofenac sodium in water samples [155]. Under visible light, H_2_O_2_-assisted Fe_3_O_4_@MIL-100(Fe) photocatalytic process 99.4% diclofenac sodium degradation was achieved. The diclofenac sodium degradation process followed Fenton-like reaction. Rasheed et al. combined MIL-100(Fe) with Fe_3_O_4_ and carbon aerogel to form a nanocomposite for the removal of tetracycline hydrochloride [156]. The comparative analysis revealed 85% antibiotic degradation by MIL-100(Fe)@Fe_3_O_4_/CA nanocomposite that was higher than observed in case of individual components. The performance of MIL-100(Fe)@Fe_3_O_4_ increased 1.6 times by coupling carbon aerogel with it. This significantly speeded up the transfer of light produced charge carriers. Further, MIL-88A (Fe) particles were immobilized onto the porous block substrate and then calcined to produce the supported MIL-88A derivative Fe_3_O_4_@ porous block substrate catalyst to remove tetracycline hydrochloride [157]. Maximum 97.5% tetracycline hydrochloride degradation was observed within 40 min of white light exposure. Even after 30 consecutive recycles, the nanocomposite could achieve very simple recycling and great reusability. According to Doan et al., a Cu_2_O/Fe_3_O_4_/MIL-101(Fe) nanocomposite degraded the antibiotic ciprofloxacin in an aqueous solution when exposed to visible light [158]. The ciprofloxacin degradation process is considerably aided by the lowered band gap energy, e^–^/h^+^ recombination suppression, and production of hydroxyl and superoxide radicals. After five cycles, the ciprofloxacin degradation efficiency dropped by just 6%, which demonstrated the outstanding cyclability of Cu_2_O/Fe_3_O_4_/MIL-101(Fe) nanocomposites. Bagherzadeh et al. reported the best performance of MIL/CoFe_2_O_4_/(3%)GO as compared to MIL-101(Fe), CoFe_2_O_4_, novel binary (MIL-101(Fe)/CoFe_2_O_4_, MIL-101(Fe)/GO and CoFe_2_O_4_/GO) for visible light photocatalytic and photo-Fenton-like degradation of tetracycline hydrochloride [159]. Besides effectively separating charge carriers and reducing their recombination, the inclusion of the optimal GO as a strong electron acceptor in MIL/CoFe_2_O_4_/(3%)GO significantly boosted the absorption of visible light. A magnetic photocatalyst Fe_3_O_4_@MIL-53(Fe) is fabricated via calcination treatment of pristine MIL-53(Fe) under visible light and composite demonstrated 99% photocatalytic removal of ibuprofen in the presence of H_2_O_2_ after 60 min [160]. In the photo-degradation process, active species like h^+^, ^•^OH, e^−^, and ^•^O_2_^−^ played crucial roles. g-C_3_N_4_@CoFe_2_O_4_/Fe_2_O_3_ composite is fabricated by taking advantage of the Z-scheme heterojunction upon combining g-C_3_N_4_ and MOF-derived CoFe_2_O_4_/Fe_2_O_3_ [161]. A small quantity of MOF-derived CoFe_2_O_4_/Fe_2_O_3_ doping significantly improved the adsorption capacity of g-C_3_N_4_ to absorb visible light and decrease its band gap. As a high-efficient mediator, the generated hybrid photocatalysts were used to activate persulfate for the degradation of emerging pharmaceutical pollutants upon exposure to visible light. After 80 min, tetracycline (99.7%), sulamethoxazole (94.8%), diclofenac (97.0%), ibuprofen (96.1%), and ofloxacin (96.5%) all could be removed. The magnetic Fe_3_O_4_@MIL-100(Fe) hybrid composites can encourage photo-Fenton-like process which has been further exploited for levofloxacin degradation [162]. The composite with a Fe_3_O_4_: MIL-100(Fe) mass ratio of 1:4 has a greater degrading efficiency (up to 93.4%) as compared to Fe_3_O_4_, MIL-100(Fe) and the other produced composites. This increased catalytic activity is attributed to the synergistic interaction between Fe_3_O_4_ and MIL-100(Fe) which favored both the effective reduction of Fe^3^^+^ to Fe^2+^ in the photo-Fenton reaction and the speedy separation of the hole-electron pair from MIL-100(Fe). Based on free radical quenching, electron paramagnetic resonance, and mass spectrometry research, a preliminary hypothesis is made on the potential degradation mechanism and intermediates of levofloxacin in the photo-Fenton reaction.

## 5. Other 2D material-based nanocomposites for treatment of pharmaceutical waste water

As potential adsorbents and photocatalyst for the efficient treatment of several environmental pollutants, functionalized two-dimensional 2D NPs/nanocomposites possess distinctive properties. The different parameters about other 2D-based nanocomposite as photocatalyst for remediation of pharmaceutical wastewater are in Table 6.

**Table 6.** 2D material-based nanocomposite as photocatalyst for remediation of pharmaceutical wastewater.

S. No.	2D-based nanocomposite	Synthesis method	Pharmaceutical contaminant or pollutant	Pollutant dose (mg/L)	Catalyst dose (mg)	Source of light; capacity	Degradation percentage	Irradiation time	Reference
1	001-T/MX	Hydrothermal	Carbamazepine	5.0	10	Solar light and UV light	98.67	180	[163]
2	Ti_3_C_2_Tx/alkalized-C_3_N_4_ (TC-aCN)	Calcination	Tetracycline hydrochloride	20	10	Xe lamp; 300 W	77	30	[164]
3	g-C_3_N_4_/Ti_3_C_2_/TNTAs	Chemical vapor deposition	Tetracycline hydrochloride	10		Xe lamp; 300 W	85.12	180	[165]
4	MXene derived TiO_2_/g-C_3_N_4_	Calcination	Tetracycline; ciprofloxacin		60	Xenon lamp; 300 W	83.5; 61.7	80; 60	[166]
5	Bi_2_WO_6_/Nb_2_CTx	Hydrothermal	Tetracycline hydrochloride	15	50	Xe lamp; 500 W	83.1	20	[167]
6	nZVI@Ti3C2-based MXene	In-situ reductive deposition	Ranitidine	5	50	UV	91.1	30	[168]
7	CuFe_2_O_4_/MXene	Sol-hydrothermal method	Sulfamethazine	40	25	Xenon lamp; 300 W	59.4	30	[169]
8	Au-GCN-MXene	Electrostatic self-assembly	Cefixime	0.5	20	Compact fluorescence light lamp; 45 W	64.69	105	[170]
9	MXene-Ti_3_C_2_/MoS_2_	Hydrothermal	Ranitidine	10	20	LED lamp; 25 W	88.4	60	[171]
10	h-BN/Bi_2_MoO_6_	Solvothermal	Tetracycline; oxytetracycline; doxycycline	20	50	Xe lamp; 300 W	99.19; 95.28; 91.04	60	[172]
11	TiO_2_–BN	Electrospinning	Ibuprofen	5	10	Halogen lamp; 150 W	34	120	[173]
12	MOF derived boron nitride nanosheet composite (BNFe-X))	Hydrothermal	Ibuprofen	10	5	Xenon lamp; 500 W	99	60	[174]
13	MoSe_2_/ZnO/p-BN	Solution-phase	Ofloxacin	30	25	Xenon lamp; 300 W	99.2	60	[175]
14	BNRU	Self-assembly and thermal	Ciprofloxacin ofloxacin enrofloxacin norfloxacin lomefloxacin	10	25	LED lamp; 9 W	100; 94.4; 87.7; 100; 94.6	15	[176]
15	Cu_2_WS_4_/YC/g-C_3_N_4_	Hydrothermal	Tetracycline	10	50	Xe lamp; 300 W	98	30	[177]
16	CdS/Au/TiO_2_	Electrochemical Anodization	Norfloxacin	5	–	Xenon lamp; 35 W	64.67	30	[178]
17	Cu_2_WS_4_/NiTiO_3_	Hydrothermal	Tetracycline	20	20	Visible light	88.6	360	[179]
18	ZnIn_2_S_4_/MoO_3_	Hydrothermal	Paracetamol	30	200	Philips halogen lamp; 500 W	87	100	[180]
19	AgInS_2_ and TiO_2_	Modified sol−gel	Doxycycline	1000	100	Mercury vapor lamp; 125 W	95	180	[181]

### 5.1. MXene nanocomposite

Several studies reported MXene as a photocatalyst for the removal of hazardous pollutants from air and water [182]. MXenes have accessible metal sites and good electron conductivity thus making it a useful co-catalyst. The hydrophilic hydrogen and oxygen groups on MXenes surface makes its interaction with diverse pollutant easy [183]. In 2D form MXenes creates Schottky barrier at the photocatalyst/MXene interface due to movement of charge from photocatalysts to the MXene. This process is helpful in inhibiting electron–hole recombination [184]. The modification of MXenes with different moieties has earlier been explored to improve its photocatalytic abilities. Faster recombination of electron holes in TiO_2_has been reported to cause low quantum yield and poor photocatalytic efficiency [185]. The combination of TiO_2_ with MXenes resulted into semiconductor heterojunctions with reduced band gap and an effective photocatalysis [186]. Shahzad et al. produced an effective photocatalytic heterostructure 001-T/MX nanocomposite using a hydrothermal method [163]. The nanocomposite has 98.67% carbamazepine degradation efficiency as compared to ~60% in case of pure Ti_3_C_2_T_*x*_ MXene. The presence of {0 0 1} facets of TiO_2_ inserted into Ti_3_C_2_T_*x*_ sheets was primarily responsible for the better photocatalytic activity of pure MXene. The superior photocatalytic activity was attributed to Schottky junctions and the additional e^−^ and h^+^ produced by {0 0 1} facets of TiO_2_. Because of free radicals ^•^OH, ^•^O_2_^–^ and the strong oxidation ability of 001-T/MX photocatalyst to breakdown carbamazepine into CO_2_ and H_2_O toward the end of the process are possible carbamazepine photocatalytic degradation mechanism. Despite good catalytic activity, the poor stability of MXenes makes them less useful. On the other hand, polymeric carbon nitride (C_3_N_4_) has good solubility being a metal-free photocatalyst, but it has disadvantages in the form of low conductivity and severe charge recombination. Keeping this in mind, delaminated-Ti_3_C_2_T_x_/alkalized-C_3_N_4_ (TC-aCN), a strong heterostructure photocatalyst made by Yi et al., was embedded into alkalized C_3_N_4_ in a few layers without being oxidized [164]. The combined effect of both these effects has made the process stable and has been used for the visible light assisted removal of tetracycline hydrochloride with 77% efficiency. The robust TC-aCN heterostructure in the form of composite having close contact between its components, Schottky junction formed between the alkalized C_3_N_4_ and Ti_3_C and holes generated in the in the VB of C_3_N_4_ on visible light exposure were mainly responsible for the catalytic breakdown of tetracycline hydrochloride. The photocatalyst demonstrated exceptional stability and reusability up to four cycles. In another study, g-C_3_N_4_/Ti_3_C_2_/TNTAs has been used as a visible-light-driven catalyst for the 85% degradation of tetracycline hydrochloride. g-C_3_N_4_ and Ti_3_C_2_ mixture had uniformly covered the TiO_2_ nanotube arrays that led to increase in surface area, better light absorption, and charge carrier migration. Hydroxylation, dimethyl amino loss, aromatic ring, double bond oxidation, and carboatomic ring cleavage were identified as five steps of the degradation process [165].

A single step in-situ calcination process has been reported to prepare MXene-derived TiO_2_/g-C_3_N_4_. The nanocomposite demonstrated higher photocatalytic degradation of tetracycline and ciprofloxacin compared to TiO_2_/graphene, g-C_3_N_4_, and conventionalTiO_2_/g-C_3_N_4_. Better photocatalytic activity was attributed to improved visible light adsorption, carrier separation, and generation of ^•^OH and ^•^O_2_^–^ free radicals [166].

Simple hydrothermal procedure has been used to produce Bi_2_WO_6_/Nb_2_CTx nanosheets [167]. Nb_2_CTx nanosheets significantly increased the separation of carriers and transfer of electrons from conduction band of Bi_2_WO_6_ to Nb_2_CTx. The created at the Bi_2_WO_6_/Nb_2_CTx interface act as Schottky junction to prevent recombination of photogenerated electron-hole pairs. Bi_2_WO_6_/Nb_2_CTx hybrid nanosheet photocatalysts have shown superior photodegradation efficiency for tetracycline hydrochloride as compared to Bi_2_WO_6_.

**Figure 6.**
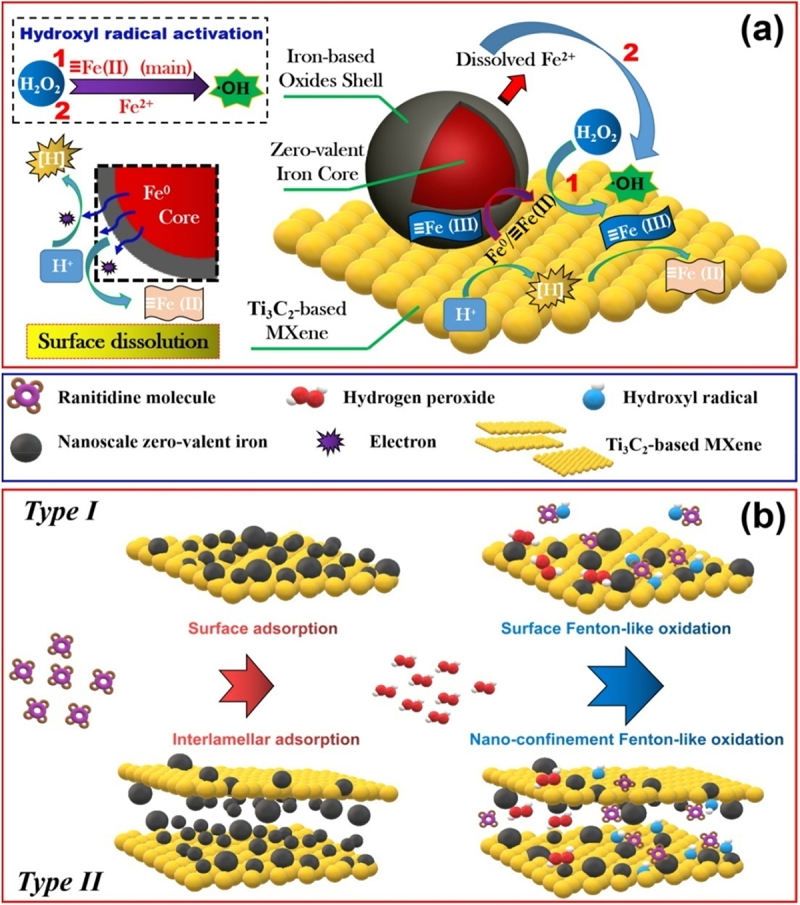
(a) Schematic illustration of the mechanism of ranitidine degradation by nZVI@Ti_3_C_2_/H_2_O_2_ system. (b) possible type of reaction zone in this heterogeneous Fenton-like oxidation system [168].

Zero-valent iron NPs functionalized Ti_3_C_2_-based MXene (nZVI@Ti_3_C_2_) nanosheets has been used for the treatment of ranitidine [168]. Nanosheets acted as Fenton like catalyst for the H_2_O_2_ dependent oxidation of ranitidine. Ti_3_C-based MXene prevented the agglomeration of and promoted the electron transmission between the magnetic NPs. Ranitidine molecules are primarily broken down by hydroxyl radical (^•^OH) attacks, particularly those that targeted surface-bound ^•^OH_ads_ (Figure 6.). Diluted hydrochloric acid treatment was effective to regenerate the catalyst for multiple times use. CuFe_2_O_4_/MXene heterojunction has been used for the photocatalytic degradation of sulfamethazine antibiotic [169]. Ternary photocatalysts Au-GCN-MXene nanocomposites has been developed using Au plasmonic NPs incorporating graphitic carbon nitride and Ti_3_C_2_ MXene to degrade cefixime antibiotic. ^•^OH produced by nanocomposite on exposure to light exposure of the helps in the breaking of the cefixime bonds. Ti_3_C_2_ ensured the prolonged availability of photoinduced carriers by preventing the reorganization of photogenerated electron holes [170]. The synergetic effect posed by 2D GCN and 2D Ti_3_C_2_ MXene nanosheet is significant from photocatalytic activity point of view. 2D GCN nanosheets acted as semiconducting photocatalyst that effectively harvests energy from visible light and 2D Ti_3_C_2_ MXene due to their increased surface are acted as electron sink. Due to the establishment of interfacial contact with GCN nanosheets, the presence of Au NPs and MXene in the nanocomposite increased the number of active sites and prevented the interaction of charge carrier. Moreover, the Au NPs plasmonic impact enhances light absorption, improving the photocatalytic efficacy of the nanocomposite. The ternary heterojunction photocatalysts made of Ti_3_C_2_/TiO_2_/BiOCl has been reported for the treatment of tetracycline. The creation of heterostructures resulted into better generation of ^•^O_2_^−^ and ^•^OH radicals and better separation efficiency of photoinduced carriers. Even after four cycles the heterojunctions exhibited good stability and activity [187]. MXene-Ti_3_C_2_/MoS_2_ composites prepared through hydrothermal process have been documented for ranitidine photodegradation [171]. The superior catalytic activity was due to the Schottky-like heterojunction of Ti_3_C_2_/MoS_2_ which improved charge carrier separation. The nanocomposite with 37.69% concentration of Ti_3_C_2_ demonstrated the best photocatalytic activity. Maximum 88.4% ranitidine degradation was achieved within 60 min.

### 5.2. Boron nitride nanocomposite

Hexagonal boron nitride (h-BN) has a van der Waals heterojunction and 2D atomic crystal structure like graphene and hence, is referred as ‘white graphene.’ It is made up of an equal amount of nitrogen and boron atoms [188]. h-BN are unique characteristics that suggest it has great potential for use in catalysis, adsorption, and energy storage [189–192]. h-BN is a hydrophobic material with increased tendency to aggregate in water; thus, considerably decreasing its ability to treat organic contaminants [193]. Grinding and surface modification of h-BN can alter its surface characteristics, increasing its sorption capacity [194].

Du et al. developed an ultrathin h-BN/Bi_2_MoO_6_ heterojunction with good photocatalytic activity using a workable solvothermal method [172]. The surface of h-BN is uniformly loaded with Bi_2_MoO_6_. The addition of h-BN boosted the photocatalytic ability through better absorb of light. Nanocomposites having 50 wt% h-BN/Bi_2_MoO_6_ had better degradation with maximum 99.19%, 95.28%, and 91.04% efficiency in case of tetracycline, oxytetracycline, respectively. h^+^ and ^•^O_2_^–^ were mainly responsible for photocatalytic activity. h-BN prevented photogenerated electron-hole pairs from recombining. TiO_2_-BN nanocomposites prepared and their potential as attractive photocatalysts for the treatment of ibuprofen in water is explored under visible light [173]. The TiO_2_-BN composites appeared to have shorter band gaps as compared to pure TiO_2_ nanoscale fibers as per UV-vis absorption spectra. Higher photocurrents are reported for the TiO_2_-BN nanocomposites than for pure TiO_2_ which suggested that negative charge on the BN nanosheets encouraged the charge separation by transporting the holes to the TiO_2_ surface. The maximum photocatalytic activity for the treatment of ibuprofen under visible light is demonstrated by TiO_2_-10 wt% BN and kinetic rate constant is almost 10 times more for TiO_2_-10 wt% BN than pure TiO_2_ [174]. The addition of boron nitride nanosheets to MIL-53(Fe) altered its morphology and giving it a distinctive prism shape for the improved persulfate assisted catalysis of ibuprofen. BNFe-3 Composites containing three percent boron nitride nanosheets exhibited maximum 99% ibuprofen degradation activity. Ibuprofen degradation was aided by the formation of ^•^SO_2_^−^, ^•^OH and ^•^O_2_^–^. Cai et al. produced MoSe_2_/ZnO/p-BN photocatalyst for the degradation of ofloxacin (OFL). Nanocomposite with 30 wt % of produced maximum 92.2% ofloxacin degradation under visible light irradiation. Synergetic effect of tight adsorption of ofloxacin by p-BN and better visible light absorption by flower-shape MoSe_2_ was responsible for improved catalytic activity. Active species quenching tests revealed that the degradation of antibiotic was largely mediated by e^−^, ^•^O_2_^−^, h^+^, and ^•^OH [175]. Boron nitride quantum dots-functionalized reduced g-C_3_N_4_ (BNRU) nanocomposite has been produced for the ciprofloxacin, ofloxacin, enrofloxacin, norfloxacin, and lomefloxacin with 100%, 94.4%, 87.7%, 100%, and 94.6% efficiencies, respectively. BNRU had a 1.7-fold greater carrier density as compared to ultrathin g-C_3_N_4_. Synergistic effects of defect engineering and quantum dots loading were mainly responsible for better antibiotic degradation activity of BNRU [176].

### 5.3. Metal chalcogenide nanocomposite

Metal chalcogenides due to their easy availability in a range of chemical composition, variable bandgap, light tuneable optical, electrical, and catalytic properties are in focus for treatment of pharmaceutical contaminant in water [195]. Hence, metal chalcogenide has been used to form 2D/2D heterojunctions to prepare better photocatalyst through separation and transportation of charge carriers [114,196]. Che et al. reported the production of yeast-derived carbon (YC) functionalized Cu_2_WS_4_/g-C_3_N_4_ heterojunction to form 2D nanocomposite. The Cu_2_WS_4_/YC/g-C_3_N_4_ has been used for the degrading tetracycline under visible light [177]. Cu_2_WS_4_ has a strong photo-catalytic activity and ease of crystal surface modulation. YC sphere acted as electron bridge which helped to prevent rapid electron recombination and significantly increased the ability to absorb visible light. The nanocomposite exhibited better catalytic activity than individual pure components. O_2_^−^, h+ and ^•^OH all have a significant impact on the degradation of tetracycline. An immobilized Z-scheme CdS/Au/TiO_2_ nanobelts composite photocatalyst made of CdS, Au, and TiO_2_ nanobelts were produced to augment the norfloxacin degradation activity on exposure to solar light [178]. The CdS NPs improved overall light harvesting, TiO_2_ NBs offered more absorption and reaction sites for the special designs and Au served as the electron transfer mediator, boosted the interfacial charge transfer and effective separation of electrons and holes. CdS/Au/TiO_2_ nanobelts showed the highest rate of degradation after being exposed to light for 60 min. The other photocatalysts degrade in the following order: CdS/Au/TiO_2_ NBs (64.67%) > CdS/TiO_2_ NBs (55.94%) > Au/TiO_2_ NBs (50.60%) > TiO_2_ NBs (42.04%). This trend is ascribed to increased light absorption and the separation of photogenerated electrons and holes result in mentioned composite. Superoxide radicals (^•^O_2_^−^) shown to be the most active species throughout the photocatalysis process in the radical trapping tests. Cu_2_WS_4_/NiTiO_3_ (CWS/NTO) composite prepared using an easy electrospinning/calcination approach along with hydrothermal procedure has been documented to show improved abilities to separate photo-generated charge carriers, endowing these composites with good and enduring photocatalytic performance for tetracycline. The degradation rate for pure NTO and CWS is just 21.5% and 43.9%, respectively within 60 min. The degradation efficiency for tetracycline exhibited a substantial improvement after the formation of CWS/NTO heterojunction under the same conditions, with degradation efficiency 88.6%, CWS/NTO composites (x = 0.5). Free radical species ^•^O_2_^−^ and h^+^ were mainly responsible for the degradation of tetracycline degradation are [179]. Z-scheme ZnIn_2_S_4_/MoO_3_ is fabricated using hydrothermal method tailed by the impregnation for the efficient degradation of paracetamol as compared to pure MoO_3_ and ZnIn_2_S_4_ [180]. The components of composite were tightly bound that is advantageous for the catalysis and ensured efficient charge carriers’ separation. ZnIn_2_S_4_/MoO_3_ has prolonged electron-hole pairs separation and the better visible light absorption owing to the direct Z-scheme system. Ganguly et al. reported ternary metal chalcogenide heterostructure nanocomposites photo-catalyst of titania via hybrid nanoarchitectures of AgInS_2_ with TiO_2_ using a modified sol-gel technique [181]. In comparison to their pristine parent samples, nanocomposites exhibit better light absorption as well as charge carrier separation. 0.5 wt% AgInS_2_ loading exhibited more than 95% sunlight assisted degradation of doxycycline in 3 h. Recent studies on boron nitride (BN) have shown that it is an effective adsorbent for organic pollutants and heavy metal ions [197–199]. The low density and sufficiently robust graphene nanoplatelet/Boron Nitride composite aerogels (GNP/BNA) have been used for the adsorption of ciprofloxacin with a capacity of 185 mg/g. Various groups on the surface of nanocomposite particularly –COO and –CO functional groups were mainly responsible for the adsorption of ciprofloxacin [200]. Further, to remove 17-ethinylestradiol from a water solution by adsorption, Xu et al. reported a magnetic MXene composite made of Fe_3_O_4_@Ti_3_C_2_ [201]. The quadratic model is used to optimize the process variables for the highest adsorption of 17-ethinylestradiol by Fe_3_O_4_@Ti_3_C_2_. Maximum 97% adsorption was achieved. Hydrogen bonding between -O, -F, and -OH functional groups on the MXene Ti_3_C_2_and aromatic ring/phenolic hydroxyl groups of 17-ethinylestradiol were mainly responsible for adsorption.

Worldwide nanotechnology market was approximately 7.73 billion in 2022 which is expected to grow up to more than $114 billion by 2030 [202]. On a rough estimate, the cost to produce one gram of gold NPs is 1600 times more than cost to produce pure gold from raw material. The cost of gold NPs may be $0.08 per microgram [203]. Industrial scale production of NPs is cheaper using bottom-up approach as compare to top-down approach. A tool CatCostTM has been used for the estimation of cost-effective precursor for the chemical synthesis of nickel catalyst. This was useful to lower nickel NPs production by 58% as estimated using the U.S. Bureau of Labor Statistics Chemical Producer Price Index [204]. Further, green synthesis routes are cheaper than chemical synthesis approach [205]. Every year more than three percent of global population is dying due to contaminated water. The problem is predicted to aggravate further with approximately more than 57% world population affected by the availability of clean drinking water. India and China account for more than 36% world population, but currently has access to only 10% of the drinking water. Countries are taking measures to clean the contaminated water. India pledged USD 46.5 billion to provide clean drinking water. Germany is one of the leading countries setting up approximately 3000 waste water treatment plants treating 920 million cubic meters of wastewater [206].

## 6. Challenges and outlooks

Advancements in medical research have resulted in the modernization of healthcare facilities, subsequently leading to a higher level of production and usage of pharmaceuticals to sustain better quality of life [207]. 26% of the world population lack clean drinking water in 2020. By 2050 half of the global urban population is expected to face clean water scarcity [208].

The presence of pharmaceutical compounds has been reported in more than 1,052 sites of 258 rivers in more than half of the world’s countries. Water pollution by pharmaceutical products is a serious health concern. Pharmaceutical pollutant has been reported to induce severe genotoxic response upon long-term exposure to human. Sub-Saharan Africa, south Asia, and South America were the most contaminated areas with under developed and developing countries were having highest amount of pharmaceutical contaminant [207,209]. Increasing access to medicines in middle income countries has led to higher volume of pharmaceuticals in wastewater as compared to low-income countries because both countries have poor waste water management while middle income countries have comparatively more access to medicines [207]. Efficient and cost-effective water treatment strategies are required by both low, as well as middle income countries. Pharmaceutical contamination of water can lead to development of antibiotic and drug resistant in microorganisms. Resistance is a natural phenomenon as these microorganisms must survive in the presence of such contaminated water. Study including bacterial antibiotic resistance for 23 pathogens and 88 pathogen-drug combinations in 204 countries and territories in 2019 covering 471 million individual records or isolates revealed that top seven microorganism accounted for four to seven hundred thousand deaths in 2019 due to antibiotic resistance. Among these methicillin resistant Staphylococcus aureus caused maximum one hundred thousand deaths in a year [209]. The aquatic animals and other organisms present in the contaminated water experience toxicity leading to ecosystem disturbance. Further, the water may be consumed by terrestrial animals and consumption of such organisms by human along with contaminated soil grown vegetables and fruits may lead to serious health hazards [210].

Among the various types of pharmaceutical wastewater treatment methods, the microorganism-based method is cost-effective and produces sludge with low toxicity to environment. However, sometimes microorganism may covert less toxic pharma waste to more toxic components. The conventional treatment methods suffer from high capital investment and require regular input to maintain efficiency. Microbial treatment methods are cost effective; generate less toxic secondary sludge and are eco-friendly [211]. Adsorption of pharmaceutical pollutant is an effective and economical strategy that allows regeneration of adsorbents. Along with routine adsorbent, the use of nano adsorbent further improves the adsorption efficiency in terms of porosity, tunability, and surface area. Currently the application of nano adsorbent is costly as compared to routine adsorbent due to the cost of nanomaterial synthesis. However, with the advent of nanomaterials that has higher adsorption capacities and can be regenerated more easily for several generations as compare to routine adsorbent overall it may be compensated for the difference in the cost [212]. The challenges with adsorbent as well as nano-adsorbent are the poor regeneration efficiency after few a cycle of usage. Different methods are different methods that are followed to regenerate the adsorbent and among them few use acid and base that ultimately pollute environment. In case of poor regeneration efficiency, the absorbent needs to be discarded and itself become a pollutant. To regenerate the nano adsorbent, the methods that have low input cost and do not use harmful chemicals to cause desorption of bound contaminant need to be thoroughly explored.

Membrane-based technologies are the most efficient available and reliable method to filter water for drinking purposes. This method is expensive and wastes more water than it filters. We still lack another reliable method that can work at large scale making it the most used method [213]. Nanomaterial functionalization of membranes has been in focus to further enhance its efficiency. The advantageous properties of nanomaterials can be completely utilized if the concerns regarding nanomaterials are seriously addressed. Although attempts are made to have strategies to handle nanomaterials more carefully than routine contaminant, however, still there is no general method for safe disposal of nanomaterials [214]. The type of nanomaterials used to prepare nano adsorbent needs to be carefully selected. Nanomaterial synthesis is challenging as the control over size, shape, and surface properties is difficult. Further, heavy metals and toxic metal-based materials should be avoided. Organic sources must be focused to prepare nanomaterials. Further the method followed to prepare organic nanomaterials must follow green chemistry principles. This would make the nano adsorbent eco-friendly itself to a great extend thus reducing the pollutant load.

Green synthesis of NPs gives better stability products however, green synthesis suffers from few drawbacks that needs to be sorted out before making it is a method of choice for large scale production [215,216]. Hence combination of environmental contamination and limited resource in case of nonrenewable resources makes recycling of nanoproducts and use of eco-friendly synthesis method essential. Grimaldi et al. designed a milli-continuous flow that has less manpower input cost by using better procedures that mainly include cleaning using mild detergent and less waste production as compare to batch synthesis method [217]. Ross et al. designed a NanoParticle Flow Synthesis System (NPFloSS) for the synthesis of large volume of silver and gold NPs [218]. Solution-based and substrate-confined nanoreactors has been used for the synthesis of various nanomaterials [219].

## 7. Conclusion

The elevated concentration of pharmaceuticals in water have posed an alarming situation for human health and environmental sustainability. This review discusses the current developments in the adsorptive and photocatalytic techniques being used to remove pharmaceutical pollutants using nanocomposites decorated with diverse types of nanomaterials. This has enhanced the effectiveness of photocatalysis and adsorption process. In-depth review is conducted on the nanocomposites that contain metal organic frameworks, carbonaceous material, metal/metal oxide and 2D materials such as MXenes, boron nitride, and metal chalcogenides. Introduction of carbonaceous QDs to the photocatalyst formulation can enhance the photodegradation rate to almost double as a result of increasing the light absorption efficiency. The degradation rate of hybrid materials can be significantly improved by using metal oxides nanomaterials. Metal oxide NPs are non-biodegradable material and can be replaced with biodegradable carbonaceous materials. Carbonaceous materials (0D, 1D, 2D, and 3D) forms hybrid material with NPs to improve the absorption efficiency up to 10 folds due to their better surface area and active functional groups. The interaction of pharmaceuticals with nanocomposites is due to coordination with unsaturated sites, π-π stacking, hydrogen, and electrostatic bond formation. Various variables involved in enhancing the photocatalytic degradation of pharmaceutical water include pH, Fenton reagent, catalyst dose, ultra violet/visible light exposure, oxidation-reduction potential, number of free radicals, and charge carrier recombination rate. This review provides critical information regarding the creation of functionalized nanocomposite materials to obtain complete or near to complete pharmaceutical waste degradation.

## Data Availability

Data sharing is not applicable to this article as no new data were created or analyzed in this study as it is a review manuscript.
